# Synthesis of Werner- and Werner-like-type clathrates, their characterization, and theoretical calculation of their properties

**DOI:** 10.55730/1300-0527.3787

**Published:** 2026-03-25

**Authors:** Zarife Sibel ŞAHİN, Zeki KARTAL

**Affiliations:** 1 Department of Energy Systems Engineering, Faculty of Engineering and Architecture, Sinop University, Sinop, Turkiye; 2 Retired Professor of Atomic and Molecular Physics, Kütahya Dumlupınar University, Kütahya, Turkiye

**Keywords:** 3-Aminopyridine (3AP), single crystal X-ray diffraction analysis, Fourier transform infrared spectroscopy, density functional theory, Hirshfeld surfaces

## Abstract

In this paper, the synthesis and structural characterization of three new coordination compounds in crystal structure consisting of a nickel atom, varying numbers of 3-aminopyridine molecules, and two isothiocyanate ion ligands are presented. The crystal structures of these compounds were determined by single crystal X-ray diffraction. Consequently, the explicit formulas and shorthand notations of the compounds are [Ni(NCS)_2_(C_5_H_6_N_2_)_2_(H_2_O)]·(H_2_O) (**1**), [Ni(NCS)_2_(C_5_H_6_N_2_)_3_]·(C_2_H_6_) (**2**), and [Ni(NCS)_2_(C_5_H_6_N_2_)_4_]·2(C_5_H_6_N_2_) (**3**). The consistency of the crystal structure data was confirmed by elemental analysis and infrared spectroscopy. These coordination compounds are Werner-like-type clathrates containing water, ethane, and 3-aminopyridine molecules as guest molecules in their structures, respectively. According to the single crystal X-ray diffraction analyses of the compounds, **1** and **3** appear to have a 3D supramolecular network-type crystal structure, while **2** has a 2D supramolecular network-type crystal structure. The N–H⋯O, O–H⋯N, N–H⋯S, N–H⋯N, and N–H⋯π bonds, as well as other bonds, contribute to the crystallization. In these compounds, the Ni atoms are surrounded by various ligand molecules, adopting an octahedral arrangement. To examine certain chemical and structural properties of the compounds, computational studies were made using their cif files using the program Gaussian 03. According to the calculation results, compound **2** has higher kinetic stability, lower chemical reactivity, and higher semiconductor properties than the other compounds. In addition, the UV–Vis and NMR excitation transitions of the compounds were calculated. Hirshfeld surface analyses indicate that C⋯H, O⋯H, S⋯H, H⋯H, and N⋯H interactions play the most significant role in the formation of their crystal structures.

## 1. Introduction

In general chemistry, compounds formed by coordination bonds between metal atoms and ligands are called coordination compounds [1–3]. The spatial orientation of the vacant orbitals in the metal atom determines the shape of the resulting coordination compound as square planar, octahedral, or tetrahedral [3,4]. The conditions of the chemical reaction that forms the coordination compound (pH, temperature, density, purity, etc.) determine whether the resulting coordination structures are simple or complex. The fact that coordination compounds exist in very different structures makes them very important industrially. If the structure of a coordination compound repeats periodically in 1D, 2D, or 3D, this compound is called a coordination polymer [5,6]. Elements form metal complexes that act as catalysts in many industrial processes. Therefore, coordination compounds are attracting more interest in both industrial and scientific fields [2,3,7]. In 1893, Alfred Werner explained the formation and structural properties of coordination compounds with his Werner theory [8]. Two important groups of coordination compounds are Hofmann-type and Werner-type compounds [1,2].

The concept of clathrate describes a structure formed when a guest molecule of appropriate size enters a porous host structure without any chemical bonding to it [8]. The general formula of Werner-type clathrates is MX_2_A_4_·nG. Here M represents a divalent transition metal cation, X an anionic ligand, A neutral ligand, G a guest molecule, and n the number of guest molecules [8–11]. Clathrates whose molecular structure formula does not exactly match the Werner-type clathrate formula are called Werner-like-type clathrates [8]. Regardless of their names or types, clathrates are very important structures due to their properties such as separating and storing specific gas mixtures, obtaining fresh water from seawater, and releasing the drugs they encapsulate at the desired time. Therefore, studying all properties of clathrates and developing new examples of clathrates are of great scientific importance. Each clathrate, formed by the introduction of molecules of varying sizes or properties into the voids within a compound with the same porous structure, possesses many different characteristics. Therefore, obtaining novel Werner-type and Werner-like-type clathrates and investigating their various properties are necessary both scientifically and for their applications. The aim of the present study was to obtain novel Werner-type and Werner-like-type clathrates with hollow crystal structures composed of nickel atoms (Ni), 3-aminopyridine (3AP), and isothiocyanate (NCS)^−^ ligands, containing various types and numbers of guest molecules in their voids. The primary goal was for water and 3AP molecules, present in the reaction medium, to enter the resulting porous structures, either individually or together. However, it was observed that an unexpected ethane molecule also appeared as a guest in one of the compounds. Furthermore, another aim was to investigate the effects of variations in the number of uncharged ligands and the types of guest molecules on various properties of the compounds.

The 3AP is a member of the aminopyridines (APs) family with the molecular formula C_5_H_6_N_2_. Its structure consists of an amino group in the meta position relative to the nitrogen atom in a pyridine ring. All APs and their various derivatives are widely used in medicine for the treatment of various diseases and in laboratories and industry for the synthesis of many new chemical compounds [11–15].

The element Ni is a transition metal with ferromagnetic properties, highly resistant to oxidation and corrosion, with very high electrical and thermal conductivity, and vital for all plants. The Ni atom can have valencies between −1 and +4 in the compounds in which it is contained. Therefore, the Ni atom can have octahedral, trigonal bipyramidal, tetrahedral, and square planar arrangements in the compounds it forms [16].

One of the most important components in the formation of Werner-type and Werner-like-type compounds and clathrates is the (NCS)^−^ ionic ligand. The (NCS)^−^ is a linear basic anion capable of forming chemical bonds from all its atoms or from the π electrons in the C≡N triple bond in the reactions it participates in [17,18].

In the present study, three different new crystalline Werner-type and Werner-like-type clathrates with the open chemical formulas [Ni(NCS)_2_(C_5_H_6_N_2_)_2_(H_2_O)]·(H_2_O), [Ni(NCS)_2_(C_5_H_6_N_2_)_3_]·(C_2_H_6_), and [Ni(NCS)_2_(C_5_H_6_N_2_)_4_]·2(C_5_H_6_N_2_) were obtained. The open chemical formulas of the compounds show that the first two compounds are Werner-like-type compounds, while the last compound is a Werner-type compound. Electrical, nonlinear optical (NLO), and thermal properties, as well as UV–Vis and NMR spectroscopies of the relevant clathrates, were theoretically calculated from cif files using the program Gaussian 03, while interatomic interactions, fingerprint maps, charge distributions, and energy types were calculated theoretically from cif files using the program CrystalExplorer.

Although the lattice (host) structures of the obtained clathrates consisted of almost identical molecules (Ni, 3AP, and NCS^−^), changes in their numbers or guest molecules (water, ethane, and 3AP) resulted in changes in their crystal systems, space groups, volumes, and some other properties.

## 2. Experimental

### 2.1. Materials

The chemical substances 3-aminopyridine (3AP; C_5_H_6_N_2_) (Sigma Aldrich, 99%), potassium thiocyanate (KSCN) (Fluka, 96%), nickel(II) acetate tetrahydrate [Ni(OCOCH_3_)_2_·4H_2_O], (Sigma Aldrich, 98%), and ammonia solution (NH_3_, Merck, 25%) were used for the Werner- and Werner-like-type clathrates that were planned to be obtained.

### 2.2. Syntheses of compounds

The general synthesis equation for the desired Werner-type and Werner-like-type clathrates can be given as follows:


(1) $$\text{m}(3\text{AP})+2(\text{KSCN})+\text{Ni}{\left({\text{OCOCH}}_{3}\right)}_{2}+\text{nG}\overset{{\text{H}}_{2}\text{O},\;{\text{NH}}_{3}}{\to }[\text{Ni}{\left(\text{NCS}\right)}_{2}{\left(3\text{AP}\right)}_{\text{m}}]\cdot \text{nG}+2[\text{K}({\text{OCOCH}}_{3})]$$

In this formula, m represents the number of uncharged ligand molecules and n represents the number of guest molecules. In the present study, the m value was gradually increased from 2 to 8. The H_2_O and 3AP molecules were expected to enter the vacancies in the structure as guest molecules.

In order to obtain new Werner- and Werner-like-type clathrates in the crystal structure, the following chemicals were used and the indicated synthesis processes were applied. For the synthesis of compound **1**, approximately 2 mmol of 3AP (0.188 g), approximately 2 mmol of KSCN (0.196 g), and approximately 1 mmol of Ni(OCOCH_3_)_2_·4H_2_O (1.798 g) were dissolved separately in a mixture of equal amounts of ethanol and twice distilled water at 55 °C with the help of a magnetic stirrer. Then one of these solutions was continuously stirred while the others were added dropwise to this mixture. The whole resulting mixture was stirred rapidly again at a constant temperature of 55 °C for 60 min. The entire mixture became a clear suspension. To make this mixture even clearer, aqueous ammonia solution was added drop by drop. The clear mixture was then rapidly stirred again for 30 min at the same constant temperature. Afterwards, this clear mixture was filtered through blue band filter paper and left to crystallize under normal laboratory conditions in a sealed environment.

For the synthesis of compound **2**, approximately 4 mmol of 3AP (0.376 g), approximately 2 mmol of KSCN (0.196 g), and approximately 1 mmol of Ni(OCOCH_3_)_2_·4H_2_O (1.798 g) were used, following the same procedure as for compound **1**.

For the synthesis of compound **3**, approximately 8 mmol of 3AP (0.752 g), approximately 2 mmol of KSCN (0.196 g), and approximately 1 mmol of Ni(OCOCH_3_)_2_·4H_2_O (1.798 g) were used, following the same procedure as for compound **1**. The crystals of each of the three compounds formed in their own unique timeframe, approximately 3 to 5 weeks.

The resulting crystals were separated from the parent liquid using black band filter paper. They were washed twice with ethyl alcohol and dried. To prevent the crystals from being affected by external conditions and deteriorating, they were placed in Eppendorf tubes and stored in a sealed container. The narrow, sharp, and intense peaks in the IR graphs of compounds provide experimental evidence of their purity level (see IR graphs of the compounds).

Since there is one Ni^2+^ ion for the 2 moles of the (NCS)^−^ ionic structure used in the preparation of the compounds and the other ligand molecules are uncharged, the compounds formed are expected to be uncharged. In the resulting compounds, the n value was observed to vary between 1 and 2 depending on the type of guest molecule.

### 2.3. Instrumentation

The FT-IR spectra of the crystalline Werner- and Werner-like-type clathrates were characterized using a Bruker Optics Vertex 70 FT-IR instrument. The concentrations of metal, carbon, nitrogen, sulfur, and hydrogen in all clathrates synthesized were determined using a PerkinElmer Optima 4300 DV ICP-OES and a CHNS-932 (LECO) elemental analyzer. Crystallographic data for the Werner- and Werner-like-type clathrates were obtained with a Bruker D8-QUEST diffractometer employing graphite monochromatic Mo-*K*_α_ (λ = 0.71073 Å).

Table 1 present both experimentally determined and theoretically calculated values for the crystalline Werner- and Werner-like-type clathrates. Strong agreement is observed between the theoretical predictions and the experimental measurements for each of the newly synthesized clathrates.

Suitable crystals of each compound were chosen for data collection using a Bruker diffractometer with graphite-monochromatic Mo-*K*_α_ radiation at 296 K. The analyses were carried out as follows: structure solution by direct methods using SHELXS-2013 [19], refinement via full-matrix least-squares using SHELXL-2013 [20], data collection with Bruker APEX2 [21], molecular visualization using Mercury [22], and structure handling through WinGX [23].

It was seen from the SC-XRD data and the elemental analysis of their structures that the closed formulas of the three new Werner- and Werner-like-type clathrates obtained in crystal form would be [Ni(NCS)_2_(C_5_H_6_N_2_)_2_(H_2_O)].(H_2_O), [Ni(NCS)_2_(C_5_H_6_N_2_)_3_].(C_2_H_6_), and [Ni(NCS)_2_(C_5_H_6_N_2_)_4_].2(C_5_H_6_N_2_). Hereafter, these compounds will be referred to as **1**, **2**, and **3**, respectively. Table 2 provides detailed information on the data collection and crystal structure determination of the Werner- and Werner-like-type clathrates. In addition, the values of some bond lengths, bond angles, N–H⋯π interactions and hydrogen-bond parameters of the Werner- and Werner-like-type clathrates are given in Tables 3 and 4, respectively.

## 3. Results and discussion

### 3.1. Crystallographic analyses of the compounds

#### 3.1.1. Crystallographic analysis of compound 1

**Figure 1:**
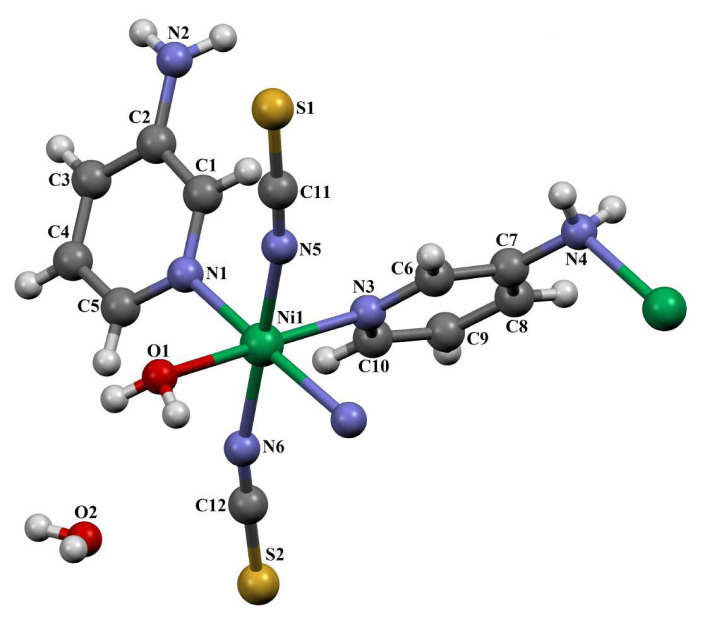
The molecular structure of **1** showing the atom numbering scheme.

The SC-XRD analysis reveals that compound **1** forms a 1D coordination polymer. Its asymmetric unit comprises a Ni(II) ion, two 3AP ligands, two isothiocyanate ions, one coordinated water molecule, and one noncoordinated water molecule (Figure 1).

Figure 1 illustrates that, in compound **1**, the Ni(II) center is coordinated by two 3AP molecules, two NCS^−^ ions, and one water molecule, each serving as a ligand. However, one water molecule that is not bonded anywhere has the character of a guest molecule. The water molecule contributed more than expected (as both a ligand and guest molecule) to the formation of this compound.

In addition, as seen in Figure 1, one of the two 3AP ligand molecules in the structure of compound **1** is bonded to the Ni(II) transition metal atoms only at the nitrogen atom in the pyridine ring, and the other is bonded to the Ni(II) transition metal atoms at the nitrogen atom of both the pyridine ring and the NH_2_ group. This situation of bonding at both nitrogen atoms is an advantageous situation rarely encountered in aminopyridine compounds. Since the structural formula of this compound does not exactly match the formula of the Werner-type clathrate, this compound is a Werner-like-type clathrate.

The Ni(II) ion is coordinated in an octahedral geometry distorted by three nitrogen atoms from 3AP ligands (N1, N3, and N4^i^)((i) 3/2−x, y+1/2, 3/2−z), two nitrogen atoms from isothiocyanate molecules (N5 and N6), and one oxygen atom from aqua ligand (O1). The Ni–N_3AP_ bond distances range between 2.112(6) and 2.210(6) Å, while the Ni–N_isothiocyanate_ bond distances are 2.028(6) Å and 2.039(6) Å. The Ni–O bond distance is 2.123(6) Å. The N–Ni–N bond angles range between 85.3(2) and 175.0(3)°, while the N–Ni–O bond angles range between 86.7(2) and 177.4(2)°. The diagonal axes of **1** have angles of 175.0(3)° and 177.4(2)°, respectively. These values numerically demonstrate the distortion in geometry. The Ni(II) ions are bridged by amino groups to form a 1D coordination polymer running parallel to the [010] direction (Figure S1).

As shown in Figure S2a, guest water molecules act as a bridge between [Ni(NCS)_2_(C_5_H_6_N_2_)_2_(H_2_O)] clusters. Thus, the combination of O–H⋯N and N–H⋯O hydrogen bonds (Table 4) produces centrosymmetric $R_{4}^{4}(24)$ rings extending parallel to the [111] direction (Figure S2a). The C9 atom acts as a hydrogen bond donor to the S2 atom at (1−x, 1−y, 2−z) in the molecule, forming a centrosymmetric $R_{2}^{2}(16)$ ring with center (1/2, 1/2, 1). Similarly, the amino N2 atom acts as a hydrogen bond donor to the S1 atom at (1−x, −y, 1−z) in the molecule, forming a centrosymmetric $R_{2}^{2}(18)$ ring with center (1/2, 0, 1/2). The amino N2 atom acts as a hydrogen bond donor to the S2 atom at (1/2−x, y−1/2, 3/2−z) in the molecule, forming a zig-zag C(9) chain running parallel to the [010] direction. Similarly, the amino N4 atom acts as a hydrogen bond donor to the S2 atom at (1−x, −y, 1−z) in the molecule, forming a C(9) chain running parallel to the [010] direction. The combination of N–H⋯S and C–H⋯S hydrogen bonds produces a 2D supramolecular network (Figure S2b). In **1**, there are no π-interactions in molecular packing. The interplay of these hydrogen-bonding interactions results in the formation of a 3D supramolecular architecture.

#### 3.1.2. Crystallographic analysis of compound 2

**Figure 2:**
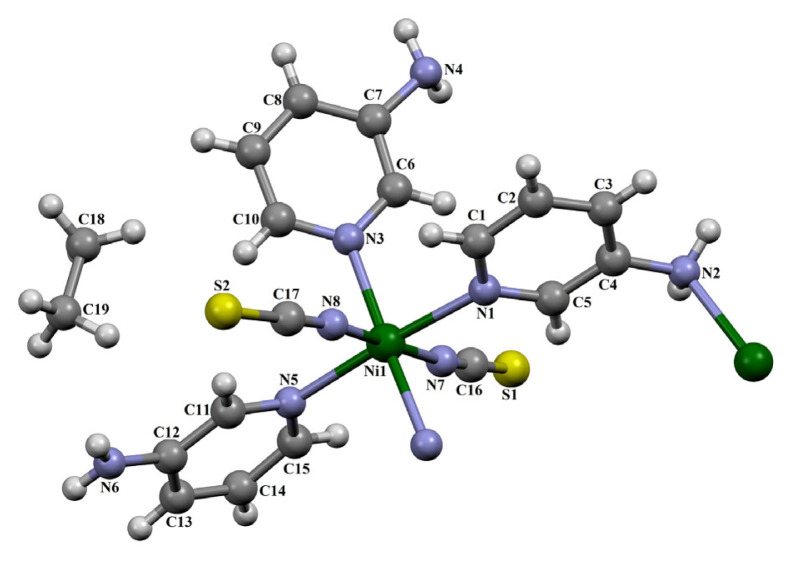
The molecular structure of **2** showing the atom numbering scheme.

The SC-XRD analysis indicates that compound **2** forms a 1D coordination polymer. There are one Ni(II), three 3AP, two isothiocyanate molecules, and one noncoordinated ethane molecule in the asymmetric unit of compound **2** (Figure 2).

In addition, as seen in Figure 2, only one of the three 3AP ligands is bonded to the Ni(II) atoms at both the pyridine ring nitrogen atom and the NH_2_ group nitrogen atom. The other two 3AP ligands are bonded to the Ni(II) atoms only at the pyridine ring nitrogen atom. Again, this is an advantageous situation rarely encountered in compounds of aminopyridines.

The ethane molecule (C_2_H_6_) that appears as a guest molecule is an unexpected type of molecule observed in the crystal structure of this compound. Under normal conditions, ethane is in the gas phase. Therefore, it is very difficult to introduce a gas molecule as a guest into the structure of any compound, and this requires a very complex and expensive vacuum experimental system for the study. The observation of the ethane molecule as a guest molecule in the crystal structure of this compound obtained under simple laboratory conditions is a great surprise. Since the structural formula of compound **2** does not exactly match the formula of the Werner-type clathrate, this compound is a Werner-like-type clathrate as well.

The Ni(II) center is coordinated by one amino nitrogen atom (N2^i^) from 3AP ligand, two nitrogen atoms (N7 and N8) from isothiocyanate groups, and three additional nitrogen atoms (N1, N3, and N5) from 3AP ligands, resulting in a distorted octahedral coordination geometry [(i) 1/2−x, y−1/2, 1/2−z]. The Ni–N_3AP_ bond distances range between 2.121 (4) and 2.203 (3) Å, while the Ni–N_isothiocyanate_ bond distances are 2.051 (4) Å and 2.062 (4) Å, respectively. The N–Ni–N bond angles range between 86.65(13) and 78.34(15)°; these values numerically demonstrate the distortion in octahedral geometry. These Ni(II) ions are interconnected via amino groups to form a 1D coordination polymer aligned along the [010] direction (Figure S3a).

The C3 atom acts as a hydrogen bond donor to the S2 atom at (1/2−x, 3/2−y, −z) in the molecule, forming a centrosymmetric $R_{2}^{2}(18)$ ring. Similarly, the amino N4 atom acts as a hydrogen bond donor to the S1 atom at (1−x, y, 1/2−z) in the molecule, forming a centrosymmetric $R_{2}^{2}(18)$ ring. The amino N2 atom acts as a hydrogen bond donor to the S2 atom at (x, y+1, z) in the molecule, forming a C(9) chain running parallel to the [010] direction. The amino N2 atom acts as a hydrogen bond donor to the N6 atom at (x, y+1, z) in the molecule, forming a zig-zag C(10) chain running parallel to the [010] direction. Similarly, the amino N6 atom acts as a hydrogen bond donor to the S1 atom at (x, y−1, z) in the molecule, forming a C(9) chain running parallel to the [010] direction. The combination of these hydrogen bonds produces a 2D supramolecular network (Figure S3b). In addition, guest ethane molecules form C–H⋯S hydrogen bonds (C19—H19C⋯S2 and C19—H19B⋯S1^i^), which contribute to the formation of a 3D supramolecular network by bridging [Ni(NCS)_2_(C_5_H_6_N_2_)_3_] clusters [(i) x, 1−y, z−1/2]. In **2**, there are no π-interactions in molecular packing.

#### 3.1.3. Crystallographic analysis of compound 3

**Figure 3:**
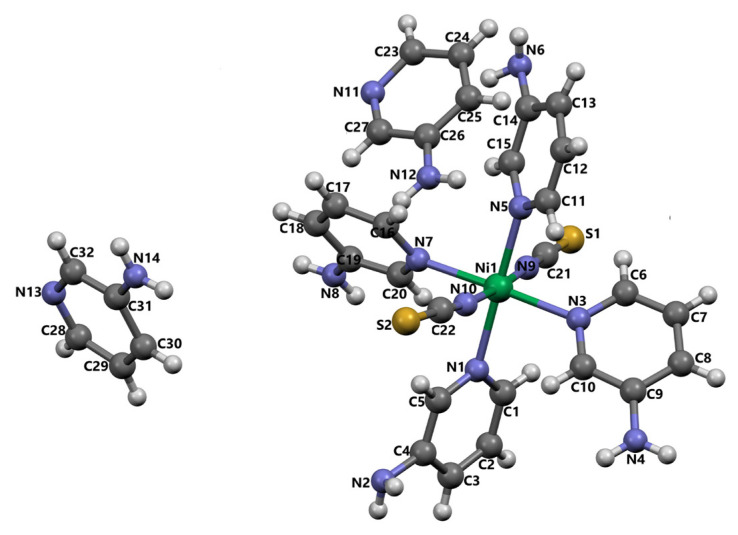
The molecular structure of **3** showing the atom numbering scheme.

In compound **3**, the asymmetric unit comprises one Ni(II) center coordinated by two isothiocyanate ligands, four 3AP ligands, and two noncoordinated 3AP ligands (Figure 3). Four 3AP ligands and two NCS^−^ ionic ligands are bonded to Ni(II) ions in a distorted octahedral geometry. In addition, there are two more 3AP molecules as guests, which are not bonded anywhere in the cavities of this compound. Indeed, the 3AP molecule contributed to the formation of this compound both as a ligand molecule and as a guest molecule. Since the structural formula of this compound exactly matches the formula of a Werner-type clathrate, this compound is a Werner-type clathrate.

The Ni(II) ion is coordinated by two nitrogen atoms [N9 and N10] from isothiocyanate molecules and four nitrogen atoms [N1, N3, N5, and N7] from 3AP ligands, thus showing a distorted octahedral coordination geometry. The Ni–N_isothiocyanate_ bond distances are 2.009(8) and 2.072(7) Å, while the Ni–N_3AP_ bond distances range between 2.120(7) and 2.159(7) Å. These bond distances compare well to the literature values with the same ligand. The N–Ni–N bond angles range between 86.7(3) and 179.2(3)°, while these values numerically demonstrate the distortion in octahedral geometry. As seen in Figure 3, all four 3AP ligands are bonded to the Ni(II) atoms at only the nitrogen atoms in the pyridine ring. Linkages between the molecules arise from a combination of N–H⋯S and N–H⋯N hydrogen bonds (Table 4). The amino N2 atom serves as a hydrogen-bond donor to the S1^iv^ atom, generating a C(9) chain aligned along the [010] direction [(iv) x, y−1, z] (Figure S4). Likewise, the N4 atom donates a hydrogen to the S1^i^ atom, producing a centrosymmetric $R_{2}^{2}(18)$ring centered at (n+1/2, n+1/2, n+1) (n = zero or integer) [(i) 1−x, 1−y, 2−z], while the amino N8 atom acts as a donor to the S2^ii^ atom, forming another centrosymmetric $R_{4}^{4}(36)$ ring centered at (n, n, n+1/2) (n = zero or integer) [−x, −y, 1−z]. Together these hydrogen bonds create edge-fused $R_{2}^{2}(18)$ rings running parallel to the [111] direction.

The combination of N–H⋯S hydrogen bonds produces an edge-fused $R_{2}^{2}(18)R_{4}^{4}(36)$ ring aligned parallel to the [111] direction (Figure S5). Moreover, there are two N–H⋯π interactions in the structure of **3** (Table 4). The N–H⋯π interactions occur between the amino groups of coordinated 3AP ligands and noncoordinated 3AP ligands of neighboring molecules (Figure S6). The combination of N–H⋯N and N–H⋯π interactions formed by the guest 3AP ligands bridges the [Ni(NCS)_2_(C_5_H_6_N_2_)_4_] clusters, creating a 3D supramolecular network.

### 3.2. Experimental FT-IR investigations of the compounds

**Figure 4:**
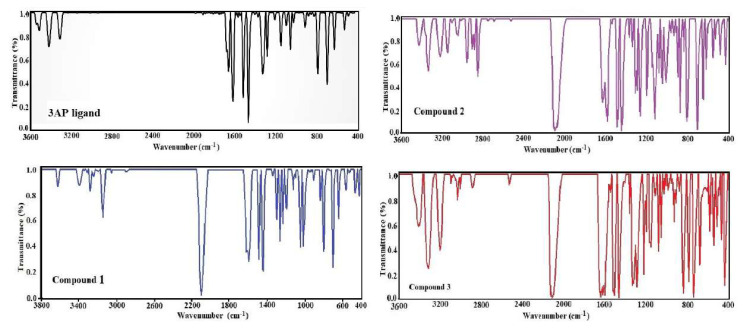
The experimental FT-IR spectra of 3AP ligand and its compounds.

The spectra of the experimental vibration frequencies recorded under normal conditions in the crystal form of the compounds are given in Figure 4. When the IR spectra of compounds are compared with the free IR spectra of each molecule that makes them up, it is observed that some peaks are displaced. This is a result of the molecules present reacting (exchanging energy) to form a new chemical compound. When new compounds are formed, changes occur in the structural and electronic arrangements of each molecule compared to their free states.

Some differences between these spectra are owing to the inclusion of multiple ligands in the frameworks of the compounds [3AP, H_2_O, and (NCS)^−^], the different number of the same ligand molecule, or the different positions of the ligands. In addition, in the comparative analysis of the compounds’ spectra, it is quite natural to see some similarities between them, because the biggest reason for these similarities is the occurrence of the same ligands and the same Ni center in the compounds. Furthermore, the fact that most of the peaks in the IR graphs of the obtained compounds are narrow, sharp, and intense is experimental evidence that the compounds are largely pure.

Experimental results for the compounds consistently demonstrate the existence of 3AP, (NCS)^−^, H_2_O ligands, H_2_O, ethane, 3AP guest molecules, and Ni atoms. The spectra of the compounds were thoroughly examined to identify the vibrational modes of 3AP, (NCS)^−^, and H_2_O ligand molecules and H_2_O, C_2_H_6_, and 3AP guest molecules.

#### 3.2.1. Experimental vibrations of the 3AP ligand molecule in compounds 1, 2, and 3

Table 5 shows the experimental vibrational modes of the free 3AP ligand and of compounds **1**–**3**, highlighting the wavenumber shifts resulting from compound formation. Examination of Table 5 shows that many vibrational modes exhibited by the 3AP are altered as a result of compound formation. Due to these effects, some vibration modes show a slight shift towards either high wavenumber or low wavenumber (+Δ or −Δ) because of the increase or decrease in the relevant coupling constant. Additionally, columns and rows indicating the frequency shifts of the ligand molecule in the compounds affected by the formation of the compounds and their sum have been added to Table 5.

These experimental vibrational data agree with those reported in earlier studies involving the same ligand molecule [11–15]. However, since the electronic charge arrangement of each compound is unique, it is very normal for the wavenumbers and shift amounts of some vibrations to have different values from each other.

In ligand molecules containing the NH_2_ group, the bonding of the N atom of the NH_2_ group to another metal atom (see Ni⋯NH_2_ bonding in compounds **1** and **2**) or the formation of hydrogen bonds between the H atoms of the NH_2_ group and the atoms of another molecule caused shifts to lower wavenumbers in the relevant stretching vibrations of both the NH_2_ group and the relevant stretching the other molecule (see NH⋯π interactions in compound **3**).

In compounds **1** and **2**, the bonding of some 3AP ligands at the N atom of the NH_2_ group to the Ni atom caused some of the electrons in this group to flow to the Ni atom. Thus, there are two NH_2_ groups with different properties in compounds **1** and **2**. This caused the splitting of some ν_as_(N–H) and ν_s_(N–H) vibrations in their IR spectra. In addition, new interactions of some hydrogen atoms in the NH_2_ group with other surrounding atoms are also one of the reasons for the splitting of these stretching vibrations.

In the formation of compounds **1** and **2**, it is clearly seen in their structural forms that some 3AP ligands bond to the Ni(II) atom at both nitrogen atoms in their structure, while in compound **3**, the 3AP ligands coordinate to the Ni(II) center exclusively through the nitrogen atom of the pyridine ring (see Figures 1, 2, and S1–S3). Spectroscopic evidence that the 3AP ligand molecule is attached at the N atom of the pyridine ring to the Ni transition metal atom in compounds **1**, **2**, and **3** is the vibrational peaks observed at wavenumbers of 429, 426, and 425 cm^−1^, respectively, in their IR spectra [14]. Similar situations have been encountered in previous studies. Another vibration peak, indicating that the 3AP ligand molecule bonds to the Ni atom at the N atom in its pyridine ring, is located in the far IR region (between 400 and 250 cm^−1^), outside the measurement range of the IR spectrometer used.

Although the 3AP ligand molecule in compound **3** only bonds at the N atom of the pyridine ring to the Ni atom, it has been observed that its ν_s_(N–H) vibration mode is also split into two (see Table 5). The reason for this splitting is that there are both 3AP ligand molecules in the compound that only bond to the Ni(II) transition metal at the nitrogen atom in the pyridine ring, and guest 3AP molecules that do not form any bonds. Another reason for the splitting of ν_as_(N–H) and ν_s_(N–H) vibrations in the IR spectrum of compound **3** is the formation of N–H⋯π interactions between the hydrogen atom of the NH_2_ group of the 3AP ligand molecules and the pyridine ring of the guest 3AP molecules in this compound.

In addition, an increase in 3AP ligand content in the compounds led to the splitting of some vibration frequencies, which was directly proportional to the number of ligand molecules (see Table 5). The reason for these splits is that some ligand molecules have different environmental arrangements.

In compounds **1**–**3**, the formation of the crystal lattice affects several key vibrational modes of the 3AP ligand, and the first five largest wavenumbers shifts (Δ) are presented below as examples:

For compound **1**: ν_as_(NH_2_) = 3394; 21, ν_s_(NH_2_) = 3279; −25, ν(C–H) = 3145; 79, δCH + ν_ring_ = 1235; 40 and δ_ring_ + NH_2_ wag + γ_C–NH2_ = 568; 21 cm^−1^.

For compound **2**: ν_as_(NH_2_) = 3432; 59, ν_s_(NH_2_) = 3339; 35, ν(C–H) = 3152; 86, δCH + ν_ring_ = 1195; 0 and δ_ring_ + NH_2_ wag + γ_C–NH2_ = 549; 12 cm^−1^.

For compound **3**: ν_as_(NH_2_) = 3448; 75, ν_s_(NH_2_) = 3209; −95, ν(C–H) = 3103; 37, δCH + ν_ring_ = 1174; −21 and δ_ring_ + NH_2_ wag + γ_C–NH2_ = 572; 35 cm^−1^.

From the examination of the total wavenumber shifts (∑Δ) in Table 5, it is seen that this value shows a direct correlation with the number of ligands. This situation appears as an experimental confirmation of the theoretical expectation.

#### 3.2.2. Experimental vibrations of the H_2_O ligand and guest molecule in compound 1

In the formation of compound **1**, the water molecule is present both as a coligand and a guest molecule. Generally, the presence of a coordinated water molecule in a compound is indicated by a broad and moderate band in the wavenumber range of 3100–3300 cm^−1^, interacting with the peaks of other molecules. The vibrational peaks of the bound (coordinated) water molecule in compound **1** interact with the asymmetric and symmetric NH_2_ stretching vibrational peaks of the 3AP molecule in this region, causing them to appear broader. Therefore, broadening was observed in the peaks of the ν_as_(N–H), ν_s_(N–H), and NH_2_ scissoring vibration modes of the 3AP ligand molecule. However, the most typical and only determining vibration frequency of the in-guest state water (or cage water) molecule can be seen very easily at 3650 cm^−1^.

#### 3.2.3. Experimental vibrations of the ethane guest molecule in compound 2

The vibrational peaks of ethane (C_2_H_6_), the guest molecule in compound **2**, are the experimental peaks at wavenumbers of 2960, 2908, 2886, and 2852 cm^−1^ in the FT-IR spectrum of this compound. To compare with these experimental results, theoretical vibration calculations were performed for the ethane molecule (see Section 4 for information). As a result of this calculation, the values of the IR active vibration modes with observable intensity were obtained as 2970, 2907, 2906, and 2856 cm^−1^. However, some theoretically calculated peaks for the ethane molecule were not observed in the experimental FT-IR spectrum because they were either IR inactive, had very low IR intensities, or the symmetry properties changed during compound formation. As can be seen here, the experimental and theoretical results have values very close to each other.

#### 3.2.4. IR active vibrations of NCS ligand in compounds 1–3

The compound KNCS is one of the most important substances used in the preparation of new compounds in organic and inorganic chemistry. For this reason, many investigations have focused on exploring its various properties [24–31]. The (NCS)^−^ ion, which has a linear structure, has three basic vibration modes that can be observed in the mid-IR region. These vibration modes are ν(NC), ν(CS) stretching, and δ(NCS) bending vibration modes. The determination and assignment of the characteristic vibration modes of the KNCS compound were performed by Jones et al. [24].

Table 6 provides a comparative overview of the characteristic vibrational modes of the (NCS)^−^ group in the compounds and in free KNCS, highlighting the effects of compound formation.

Analysis of Table 6 reveals that the characteristic ν(NC) vibration of KNCS, originally observed at 2036 cm^−1^, shifts to 2108, 2094, and 2102 cm^−1^ in compounds **1**, **2**, and **3**, respectively. These results indicate that the ν(NC) mode is influenced by compound formation, exhibiting upshifts of 72, 58, and 66 cm^−1^, respectively.

Based on its wavenumber, this vibration mode falls within the triple-bond region. The bonding characteristics of the CN groups are further supported by the bond lengths observed in the compounds. Crystal structure analysis shows that the NC bond lengths in the isothiocyanate ions are close to an average of 1.135 Å, while the Ni–N bond lengths average around 2.104 Å. These findings indicate that the ν(NC) vibration mode in the isothiocyanate groups exhibits a character very similar to that of a triple bond.

An additional vibrational mode of KNCS is the ν(CS) stretching vibration mode between carbon and sulfur atoms observed at a wavenumber of 740 cm^−1^. As expected, the formation of the compounds alters this vibrational mode as well. The ν(CS) stretching vibration mode was observed at wavenumbers of 834, 869, and 879 cm^−1^ in the new crystal structured compounds obtained, respectively. The shift amounts of this vibration mode to higher wavenumbers in the compounds are 94, 129, and 139 cm^−1^, respectively (see Table 6).

The other fundamental vibrational mode of the free KNCS compound is the δ(NCS) bending mode and its overtone, the 2δ(NCS) vibration mode. Of these vibrational modes, δ(NCS) appears as two degenerative peaks at wavenumbers of 474 and 484 cm^−1^, respectively, in the IR spectrum of KNCS, while the 2δ(NCS) overtone appears as a weak intensity peak at a wavenumber of 957 cm^−1^. Nevertheless, these vibrational modes are also influenced by compound formation. The δ(NCS) bending mode was observed as a single mode at wavenumbers of 469, 474, and 475 cm^−1^ in the compounds, respectively. The 2δ(NCS) vibration mode was observed as a weak intensity peak at wavenumbers of 909, 934, and 900 cm^−1^, respectively (Table 6).

## 4. Theoretical computational studies on the compounds

**Figure 5:**
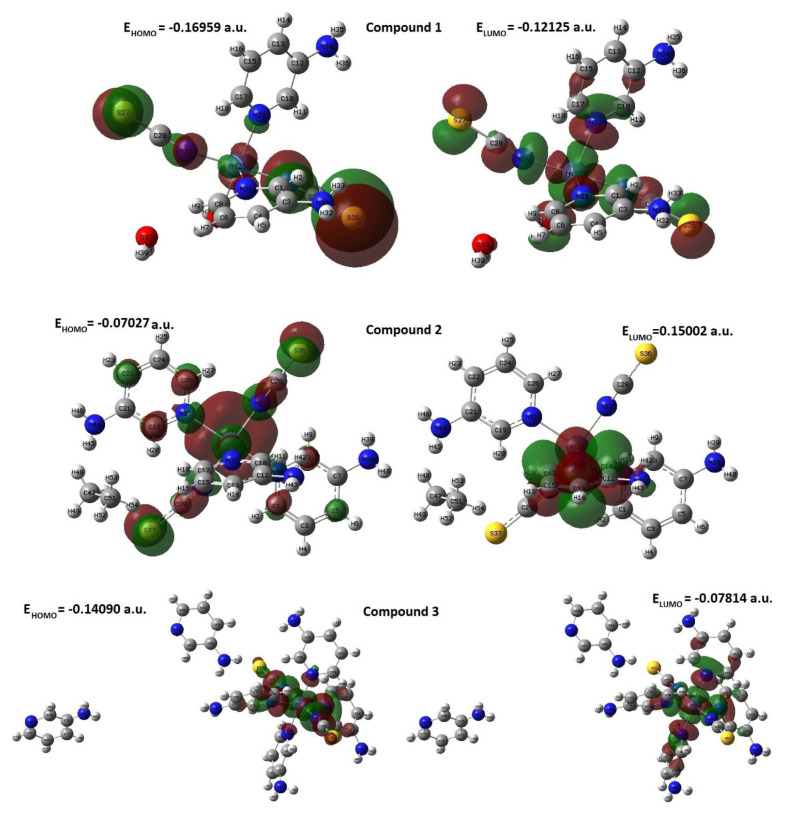
The HOMO and LUMO energy graphs of compound **1**, **2**, and **3**.

**Figure 6:**
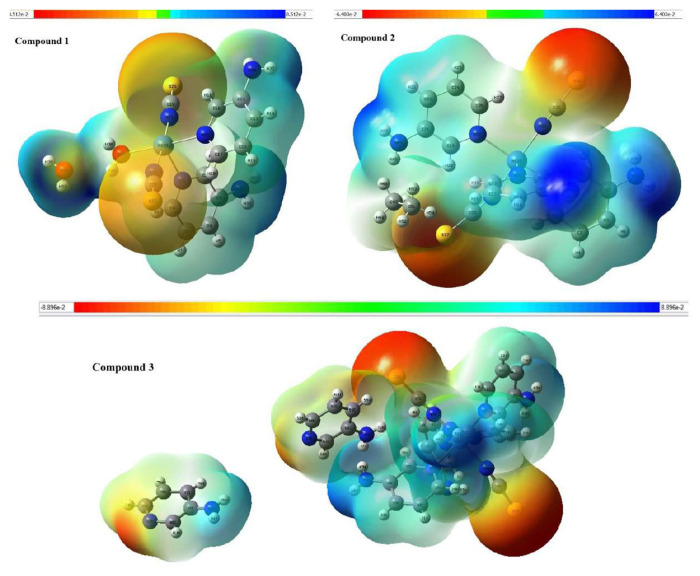
MEP maps of compounds **1**–**3** calculated at the DFT/B3LYP/LanL2DZ base set.

There is a Ni transition metal atom in the structure of all compounds obtained in crystal form. Therefore, the structural parameters of all three compounds were calculated using the program Gaussian 03 [32] and the DFT/B3LYP method [33–36] in the LanL2MB basis set. It is stated by various researchers that the LanL2MB basis set gives more consistent results than other basis sets in the theoretical studies of compounds containing transition metals in their structures. Some electronic, magnetic, and thermochemical properties of all compounds were calculated from the theoretical Gaussian values obtained [32]. Visualization of the calculated properties for all the compounds was performed using the program GaussView 4.1 [37]. The atomic numbering used in the calculations is shown in Figures 5 and 6.

The various values in the tables obtained from theoretical calculation studies of the compounds were calculated by taking data from the log files of the relevant compounds generated by Gaussian 03 using the ZEKA utility and the necessary formulas [38].

### 4.1. Mulliken atomic charge values of the ligands and compounds

The Mulliken charges defined for a compound are the electric charges that each atom of that compound has when it is in electrostatic equilibrium. The positions and amounts of Mulliken charges determine the electromagnetic properties of the compound in question. To observe the effect of compound formation on the values of Mulliken charges, the Mulliken electric charge values of the atoms of the ligands in the free state and in the compound are given in Table 7 in terms of free electron charge (e).

Analysis of Table 7 yields the following results:

➢ The atoms forming all compounds have had a certain amount of change in their charge values compared to their free states due to their contributions to the compound formation.
➢ After the formation of compounds, the electrical charge sign of some atoms remains the same while only their charge values change. Some examples of such atoms are as follows: in compound **1**: H16, H18, N23, N24, and O37; in compound **2**: H2, H4, C17, H23, N31, N38, C47, C51, and H54; and in compound 3: H11, H29, N41, N46, N49, N50, H61, C62, H74, and N83. After the formation of compounds, both the sign and the value of the electric charge of some atoms change. Some examples of such atoms are as follows: in compound **1**: C5, C19, C22, S36, and S37; in compound **2**: C5, C19, C22, S36, and S37; and in compound **3**: C1, C5, C8, C10, C14, C17, C19, C23, C26, S52, and S53.
➢ The electrical charge values of some atoms have undergone very large changes resulting from the formation of compounds. These atoms are as follows: in compound **1**: C13, Ni25, S26, and S27; in compound **2**: N33, N34, Ni35, S36, and S37; and in compound **3**: Ni51, S52, S53, C67, and C80.

### 4.2. HOMO–LUMO energy levels of the compounds

All atoms have atomic orbitals with various energy values on which electrons are most likely to be located. When a compound is formed because of the interaction of two or more atoms, the atomic orbitals also fuse to form molecular orbitals.

The highest energy level of the filled molecular orbitals is called the HOMO, and the lowest energy level of the completely empty ones is called the LUMO. The energy difference between HOMO and LUMO is an indicator of the chemical stability of that compound and the colors of its solutions. HOMO–LUMO energy graphs of the compounds were obtained using the DFT/B3LYP method with the LanL2MB basis set [25–28]. The visual representation of the HOMO–LUMO graphs is presented in Figure 5.

It can be observed that the charge and energy configurations of the HOMO state of compound **1** are generally distributed on the SCN–Ni–NCS linear structure. It is seen that the charge and energy configurations of the LUMO state of the same compound are distributed in a new form on the SCN–Ni–NCS linear structure, and on the N atom bonded to the Ni atom of the 3AP ligand molecule and the O atom bonded to the Ni atom of the water ligand molecule. Some minor ordering effects of the HOMO and LUMO states are also observed on some other atoms of the 3AP ligand molecules (see Figure 5).

Similarly, the charge and energy configurations of the HOMO state of compound **2** are generally distributed on the SCN–Ni–NCS linear structure. However, there are also small amounts of charge and energy configurations in some atoms of the other 3AP ligand molecules. It is seen that the charge and energy configurations of the LUMO state of the same compound are distributed in a new way only on the atoms of one of the 3AP ligand molecules (see Figure 5).

The charge and energy configurations of the HOMO state of compound **3** are generally seen on the SCN–Ni–NCS linear structure and on some atoms of the 3AP ligand molecules close to the Ni atom. The charge and energy distribution is highest on the Ni atom and lowest on the S atom. The charge and energy configurations of the LUMO state of the same compound are distributed more densely and in a new way on the N–Ni–N linear group and on the atoms of the four 3AP ligand molecules closest to the Ni atom (see Figure 5).

Theoretical molecular orbital calculations of all compounds were performed according to the DFT/B3LYP method [33–36] and LanL2MB basis set. According to the result of this calculation, there are a total of 137 orbitals in compound **1**, of which 88 are filled and 49 are completely empty. There are a total of 195 orbitals in compound **2**, of which 127 are filled and 48 are completely empty. There are a total of 287 orbitals in compound **3**, of which 178 are filled and 109 are completely empty.

Equations (2)–(6) present the formulas used to theoretically compute the electrochemical properties based on their HOMO and LUMO values [15,39–44].


(2) $$\Delta \text{E}={\text{E}}_{\text{LUMO}}-{\text{E}}_{\text{HOMO}}(\text{Energy gap value})$$

(3) X=(I+A)/2=-μ         (Electronegativity, Negative chemical potential)

(4) η=(I-A)/2         (Chemical hardness)

(5) S=1/2η         (Chemical softness)

(6) $$\omega =\mu^{2}/2\eta \;\;\;(\text{Electrophilicity index})$$

These values calculated theoretically from Equations (2)–(6) are given in Table 8 in eV terms.

The compounds exhibit HOMO–LUMO energy gaps (ΔE) in the descending order of ΔE_2_ > ΔE_3_ > ΔE_1_. According to this result, compound **2** has higher kinetic stability, lower chemical reactivity, and higher semiconductor properties than the other compounds.

The electronegativity values of the compounds are ranked from largest to smallest as χ_1_ > χ_3_ > χ_2_, while the chemical hardness values are ranked from largest to smallest as η_2_ > η_3_ > η_1_. The chemical softness and electrophilic index values are ranked from largest to smallest as S_1_ > S_3_ > S_2_ and ω_1_ > ω_3_ > ω_2_, respectively. In addition, considering that compound **1** has the highest chemical softness value (S) and the lowest chemical hardness value (η), it can be expected that the intramolecular charge transfer will be the highest in this compound.

### 4.3. Theoretically calculated NLO properties of the compounds

In a compound, the electric dipole moment (*μ⃗*) describes how electric charges are distributed across the molecular structure. The dipole moment of a compound is a three-dimensional vector quantity in its most general form. If the intensity or orientation of the dipole moment of a compound changes with time, the electric charges in that compound are in motion. The three-dimensional orientation of *μ⃗* in a compound is determined by the spatial arrangement of its positive and negative charge centers. The *μ⃗* of a compound remains constant under electrostatic equilibrium conditions. In this case, the position of that compound is definite and does not change.

When an external electric field is applied to an electrostatically balanced compound, the charges within the compound move, disrupting the overall charge balance. A new electrostatic equilibrium occurs in the compound. The quantity that describes the distribution of electric charges in a compound is called the polarizability (α). The most commonly used polarizability definition for a compound is usually the term average polarizability (α_0_).

In addition to the electrical properties of a compound such as μ and α_0_, there are also other electrical properties denoted by the symbols Δα, β_0_, and γ [39–44]. The components of all electrical properties of the compounds were theoretically calculated using the LanL2MB basis set with the DFT/B3LYP method. The formulas given in Equations (7)–(14) were used to calculate the μ, α_0_, Δα, β_0_, and γ values of all the compounds, respectively. All calculated values are given in Table 9 in terms of electrostatic units (esu) using the necessary conversion factors [45,46].


(7) $$\mu =\surd \mu_{x}^{2}+\mu_{y}^{2}+\mu_{z}^{2}\;\;\;(\text{Dipole moment})$$

(8) $$\alpha_{0}=\frac{\alpha_{xx}+\alpha_{yy}+\alpha_{zz}}{3}\;\;\;(\text{Mean polarizability})$$

(9) $$\Delta \alpha =\sqrt{\frac{{\left(\alpha_{xx}-\alpha_{yy}\right)}^{2}+{\left(\alpha_{yy}-\alpha_{zz}\right)}^{2}+{\left(\alpha_{zz}-\alpha_{xx}\right)}^{2}+6(\alpha_{xy}^{2}+\alpha_{xz}^{2}+\alpha_{zy}^{2})}{2}}\;\;\;(\text{Anisotropies of polarizability})$$

(10) $$\beta_{x}=\beta_{xxx}+\beta_{xyy}+\beta_{xzz}\;\;\;(\text{x component of }\beta_{0})$$

(11) $$\beta_{y}=\beta_{yyy}+\beta_{xxy}+\beta_{yzz}\;\;\;(\text{y component of }\beta_{0})$$

(12) $$\beta_{z}=\beta_{zzz}+\beta_{xxz}+\beta_{yyz}\;\;\;(\text{z component of }\beta_{0})$$

(13) $$\beta_{0}=\sqrt{\beta_{x}^{2}+\beta_{y}^{2}+\beta_{z}^{2}}\;\;\;(\text{First-order static hyperpolarizability})$$

(14) $$\gamma =\frac{1}{5}\{\gamma_{xxxx}+\gamma_{yyyy}+\gamma_{zzzz}+2[\gamma_{xxyy}+\gamma_{xxzz}+\gamma_{yyzz}]\}\;(\text{Second-order static hyperpolarizability})$$

According to the values in Table 9, the ranking of the values of some properties of the compounds according to the relationship between them is as follows:

➢ *μ*_2_ ≅ 1.24*μ*_3_ ≅ 1.37*μ*_1_
➢ Δ*α*_3_ ≅ 1.28Δ*α*_1_ ≅ 1.84Δ*α*_2_
➢ *α*_0_1__ ≅ 0.80*α*_0_2__ ≅ 0.47*α*_0_3__
➢ *β*_0_3__ ≅ 15.76*β*_0_1__ ≅ 14.53*β*_0_2__
➢ *γ*_3_ ≅ 7.43*γ*_1_ ≅ 5.04*γ*_2_

While there is not much difference between the values of some electrical properties of these compounds (e.g., μ, Δα, and α_0_), there are quite large differences, especially between the first-order and second-order static hyperpolarizability values (e.g., β_0_ and γ). According to these comparison results, compound **3** stands out due to its Δα, β_0_, and γ values, with compound **1** having the highest α_0_value and compound **2** the highest μ value.

In addition, some thermochemical properties of the compounds in question, which determine their interactions with other atoms and molecules in their environment, were calculated with Gaussian 03 [32] and the results given by the ZEKA utility [38] are shown in Table 10.

From a careful examination of Table 10, it is seen that the thermochemical properties of the compounds, i.e. E, C_v_, S, and E_v0_ values, change directly proportional to the number of atoms in the compound, and the rotational constants vary inversely with the number of atoms.

### 4.4. Molecular electrostatic potential (MEP) of the compounds

The reactive regions found in the structures of compounds are indicated by their MEP maps. MEP maps of compounds **1**–**3** were created using the B3LYP/LanL2DZ method (Figure 6). In a MEP map, the red and yellow areas indicate the areas with negative electrostatic potential energy, while the blue and white areas represent the low electron density and partially positive region. Negative (red and yellow) regions in a compound’s MEP map are associated with electrophilic reactivity, while positive (blue and white) regions are associated with nucleophilic reactivity. According to Figure 6, negative regions, typically found around sulfur atoms in compounds, are the regions with the highest electron density and high electronegativity. In contrast, the blue regions observed near hydrogen atoms bonded to carbon indicate potential nucleophilic sites. The distinct spatial separation between the positive and negative potential regions suggests a significant polarity within the molecular framework. Additionally, the prominent blue regions near the amine hydrogens highlight possible nucleophilic centers that may participate in hydrogen bonding or proton transfer processes, indicating a more complex reactive profile.

### 4.5. Theoretical UV–Vis spectra calculated from crystal structure data of the compounds

**Figure 7:**
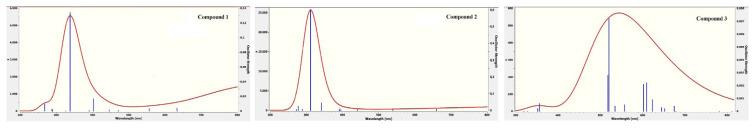
Theoretical UV–Vis absorption spectra of the compounds in water solvent.

Theoretical UV–Vis absorption spectra of the compounds in question (for N_state_ = 20) were analyzed under [scrf = (iefpcm, solvent = water)] conditions with the theoretical basis set TD-SCF, B3LYP/LanL2MB. Electronic transitions of the compounds corresponding to 20 excitation levels in the UV–Vis energy range were calculated. The UV–Vis graphs of the compounds were obtained from the relationship between their theoretically calculated wavelengths (λ, in nm) and their oscillation strengths (f, without units) (see Figure 7) [47–49].

For each compound, the theoretically calculated number of excitations corresponding to N_state_ = 20 occurred in the wavelength range of approximately 200 to 2500 nm, depending on the structural and electronic properties of the compounds. Theoretically calculated UV–Vis excitation transitions for the compounds at N_state_ = 20 and their related information are given below.

For compound **1**, a total of 95 theoretical excitation transitions occurred, all of which were observable. For compound **2**, a total of 94 theoretical excitation transitions occurred. While 85 of these were observable, 9 were forbidden excitation transitions. For compound **3**, a total of 70 theoretical excitation transitions occurred. While 62 of these were observable, 8 were forbidden excitation transitions. All UV–Vis excitation transitions occurring during the formation of compounds, regardless of their properties, play a crucial role. In Table 11, only the first five theoretically calculated excitations with the largest contribution to the UV–Vis excitation transitions of the compounds are listed for illustration purposes.

Generally, transitions between approximately 210 and 290 nm in the UV–Vis excitation transitions of compounds indicate the π → π* and n → π* transition types. Transitions between approximately 300 and 400 nm can be considered to correspond to the L → M charge transitions between ligand atoms and the transition metal, and transitions between approximately 400 and 800 nm can be considered to correspond to the interaction of radiation in the visible region with the compounds [47–49].

From a careful examination of Table 11, the following conclusions are evident:

➢ π → π* and n → π* transitions were observed in compound **1** at wavelengths of 267.78 and 289.17 nm, and in compound **2** at wavelengths of 272.54, 277.54, and 288.91 nm. However, no such transition was observed in compound **3**.
➢ L → M charge transitions were observed in compound **1** at a wavelength of 390.56 nm, in compound **2** at a wavelength of 395.84 nm, and in compound **3** at wavelengths of 351.17 and 355.26 nm.
➢ The visible region transitions were observed in compound **1** at a wavelength of 470.70 nm, in compound **2** at a wavelength of 440.88 nm, and in compound **3** at wavelengths of 598.66 and 605.64 nm.

The transition with the highest C_i_ coefficient for each compound is shown in Figure S7 as an example. These example transitions are 79 → 89 for compound **1**, 112 → 114 for compound **2**, and 178 → 181 for compound **3**. These allowed transitions with the highest C_i_ coefficient correspond to the visible region transitions in compounds **1** and **3** and to the π → π* and n → π* transitions in compound **2**.

### 4.6. Theoretical NMR analysis of compounds 1, 2, and 3

The chemical and magnetic shift values of all elements in the theoretical and experimental NMR spectrum of any compound, especially the ^1^H and ^13^C shift values, are the greatest evidence of structural changes occurring in that compound [50–52]. By interpreting either theoretical or experimental shift values, explanatory and supportive information about the structure of the compound is obtained. Using the crystal structure data of the three compounds, their NMR spectra were obtained in Gaussian 03 with the help of the same basis sets used to acquire these data. The most popular GIAO approach was chosen for calculating the NMR spectra of the compounds [50–53].

The isotropic chemical shift for any element in a compound is determined by the structural electronic environment of the nuclei that produce that signal. This value is independent of the location of the element in the compound. When an external magnetic field interacts with a compound, the observed charge in the resonance position of a specific atom is referred to as anisotropic displacement. The magnitude of this shift displacement varies depending on the spatial environment of the atom within the molecular structure.

The isotropic chemical shift values of the elements indicated with the same symbol between −0.05 and +0.05 ppm were included in the same group and an average isotropic shift value was assigned to them. The number of atoms opposite the average isotropic shift value of any group indicates the degeneracy number of that group. The value of anisotropic shift depends on the position of the element in the compound and the orientation of the external magnetic field there, i.e. it is a vector quantity. For this reason, anisotropic shift values cannot be averaged like isotropic shift values.

If the NMR spectrum of a compound is recorded in a solvent, either experimentally or theoretically, the average isotropic shift values obtained are calibrated by taking the peak value in the NMR spectrum of tetramethylsilane (TMS) as zero. There are some substances that can be used as reference materials other than TMS. However, the most suitable reference material in terms of chemical and physical properties is TMS [54].

The isotropic average chemical shielding values obtained from the NMR spectra of the compounds were rearranged using the B3LYP/6-311+G(2d,p) GIAO method, with respect to the TMS reference material for the C and H elements and the NH_3_ reference material for the N element [32,55].

**Figure 8:**
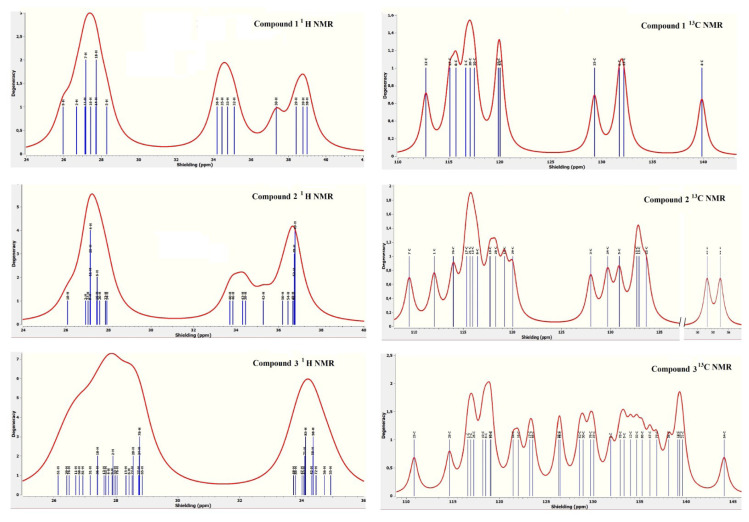
The ^1^H and ^13^C NMR spectra of compounds **1**–**3**.

To provide examples of the NMR spectra of the compounds, their theoretical ^1^H and ^13^C graphs, drawn by Gaussian 03 according to their average chemical shielding values, are given in Figure 8.

Some of the theoretical NMR results obtained for compounds **1**, **2**, and **3** are listed in Table 12 for illustrative purposes. The following conclusions can be obtained from examination of the NMR spectra of the compounds:

➢ Various elements in a compound may have positive or negative chemical shielding values depending on their location, interactions with the environment, and charge status (see N, O, Ni, S, C, and H elements in the compounds).
➢ The elements with the greatest shift toward positive or negative chemical shielding values in the compounds are Ni and O in compound **1** and Ni and N in compounds **2** and **3**.
➢ The degeneracy numbers of H and C elements in the compounds varies between 1 and 4.
➢ Compounds **1**, **2**, and **3** are formed by proton groups consisting of 37, 47, and 75 same type atoms with different properties, respectively.
➢ The chemical shielding value of the O element (in the ligand molecule), which is connected to the Ni atom of the H_2_O molecules in compound **1**, is larger than the chemical shielding value of the O element (in the guest molecule) that has not bonded.
➢ In these compounds, the chemical shielding value of N atoms is generally ranked from least to most as follows: N_pyridine_, N_isothiocyanate_, and N_amino_.
➢ The chemical shielding values of the S atoms in these compounds are almost equal. This indicates that the environmental properties of the S atoms in these compounds are similar.
➢ In compound **3**, the chemical shielding values of the N elements in the 3AP ligand molecules are greater than those of the N elements in the guest 3AP molecules.
➢ Ni is the element that has a negative chemical shielding value in all three compounds.

### 4.7. Hirshfeld surface analyses of the compounds

Both long-range and short-range interactions are crucial in the crystallization process. The intensities of short-range interactions are considerably smaller than those of long-range interactions. Spackman et al. developed a program called CrystalExplorer that calculates short-range interactions using a cif file of a compound [56]. This program calculates various surface types and properties of a molecular structure of interest [56]. Necessary information about CrystalExplorer and the properties of the surfaces it calculates is available at various sources [15,56]. A 3D Hirshfeld surface corresponding to some specific values of a crystal structure from the cif file is generated by CrystalExplorer. In addition, a 2D map of this 3D Hirshfeld surface, called the fingerprint map of the compound in question, is generated by CrystalExplorer. From a fingerprint map, very important information is obtained about the interactions between neighboring atoms of the molecular structure of that compound [56]. However, although CrystalExplorer cannot analyze the molecular structures of polymeric crystals, it is quite successful in calculating and visualizing the voids, radii of curvature, and surface values of the voids in crystalline molecular structures [56]. On a Hirshfeld surface, the red, blue, and white regions correspond to hydrogen-bond interactions, long-range interactions, and van der Waals interactions, respectively [56]. The color intensity on the surface is directly related to the strengths of the corresponding interactions. As a graphical tool, Hirshfeld surface analysis provides an effective means of visualizing and interpreting different types of interactions within a molecular structure [56].

**Figure 9:**
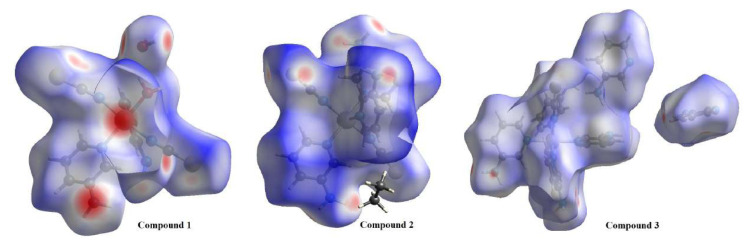
Views of the 3D Hirshfeld surfaces of the basic structures of the compounds from different angles.

**Figure 10:**
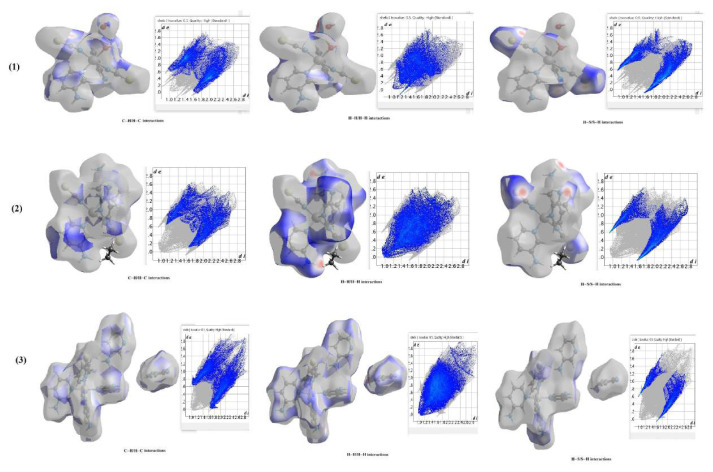
2D fingerprint plots of the three highest-valued interatomic contacts of the compounds.

The d_norm_ states, Hirshfeld surfaces, fingerprint plots, various intermolecular contacts, and crystal void values generated by CrystalExplorer for the compounds in question are given in Figures 9 and 10 and Table 13. Although the necessary calculations could be performed by incorporating the guest molecules in compounds **1** and **3** into the Hirshfeld surface using CrystalExplorer, a calculation incorporating the guest molecule in compound **2** into the Hirshfeld surface could not be carried out in any way.

It is clearly seen from Table 13 that the interactions between atoms that contribute the most to the formation of the crystal structures of the compounds are C⋯H, H⋯H, and H⋯S and the interactions between atoms that contribute the least are Ni⋯H, N⋯N, N⋯S, and S⋯S.

As shown in Table 13, the C⋯H/H⋯C, H⋯H, H⋯S/S⋯H, and H⋯N/N⋯H interactions in all three compounds have the highest values and are responsible for the formation of their supramolecular crystal structures of various orientations (1D, 2D, or 3D).

However, in addition to these interactions between atoms that contribute the most to the formation of the crystal structures of compounds, the interactions whose contributions are close to zero are as important as the interactions that contribute the most. As a result of all the interactions, the crystal structure of the compound becomes the most stable.

A careful examination of the fingerprint graphs of the compounds (Figure 10) and Table 13 shows that the interactions that contribute most to their formation are, from largest to smallest, 1 > 3 > 2 for C⋯H contacts, 1 > 2 > 3 for H⋯S contacts, and 3 > 2 > 1 for H⋯H contacts.

**Figure 11:**
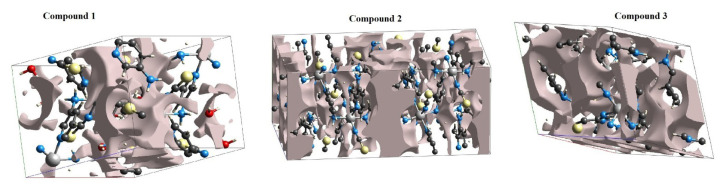
3D views of the crystal voids in the structure of compounds in different views.

In addition, the crystal voids formed in the structure of each compound were also calculated by CrystalExplorer. Figure 11 shows the crystal voids formed in the unit cells of the compounds in 3D. The volumetric values of these voids correspond to the sum of the global atomic electron densities around its atoms for each compound.

For a clearer comparison of the void properties of each compound, the data are presented collectively in Table 14. Examination of this table reveals that the void values decrease in the order 2 > 3 > 1.

The value that defines the strength in a crystal structure is a measure of the response of that crystal to mechanical effects applied to it from outside or inside. If a crystal structure has a very large empty volume, the crystal structure in question has a weak packing form between the molecules that form it. In other words, that crystal structure is not strong enough. It can easily break apart under the influence of a small external or internal mechanical force. If a crystal structure is to be used especially for gas and other guest molecule storage, its mechanical stability must have a sufficiently large value.

When the volume values of the voids in the crystal structures of the compounds in the present study are compared with those of the voids in the crystal structures of the compounds in our previous study [15], it is seen that the present values are larger. This result shows that the newly obtained compounds are more suitable than the old ones in terms of gas and other guest molecule storage properties.

**Figure 12:**
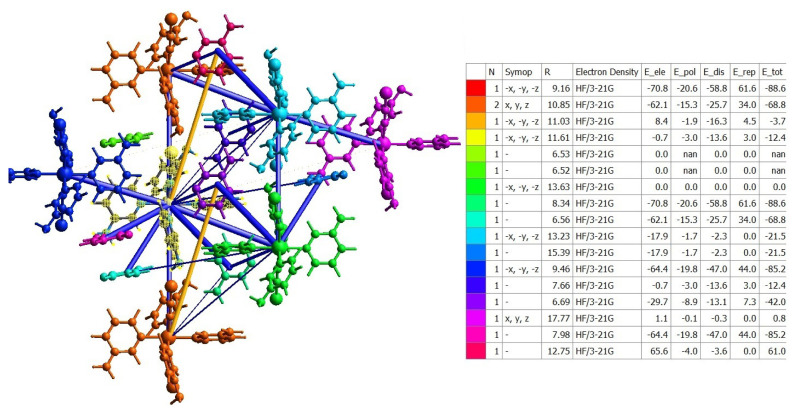
Figure and table of interaction energies of compound **3** calculated with the HF/3-21G model. In this figure, the central molecular structure is highlighted with a yellow mesh.

As is known, CrystalExplorer cannot calculate the interaction energies of compounds with polymeric structures. Since compound **3** contains 84 atoms and its interaction energies require considerably more time than the DFT/B3LYP method allows, this calculation was made using the DFT/HF method with the 3-21G basis set, which is less time consuming. Therefore, only the interaction energies of compound **3** were calculated and the result is given in Figure 12.

According to Figure 12, the two interactions with the most dominant total energy value in the formation of compound **3** are the interactions between the green and red groups located at 8.34 and 9.16 Å from the central molecular structure, respectively; these values are both −88.6 kJ/mol. In addition, according to Figure 12, the second two interactions with the most dominant total energy value in the formation of compound **3** are the interactions between the central molecular structure and the blue and pink groups located at 7.98 and 9.46 Å from it, respectively; these values are both −85.2 kJ/mol.

## 5. Conclusion

In the present study, two Werner-like-type and one Werner-type clathrate were synthesized in crystalline form for the first time. Each clathrate contains different molecules as guest molecules: H_2_O, C_2_H_6_, and 3AP. When performing studies on Werner-type and Werner-like-type clathrates, it is quite difficult to find a large number of examples. Furthermore, the structures used as guest molecules are quite small and limited in number. The experimental SC-XRD, elemental analysis, and IR results of the compounds, along with the theoretical calculations derived from the data in their cif files, support the presence of functional groups in the compounds and their chemical contribution to their formation. Compounds **1** and **3** have supramolecular network crystal structures formed by various interactions in 3D, while compound **2** has a similar structure in 2D. Considering all the properties of the compounds, compound **3** ranks first with the highest values in six categories: molecular mass, β_0_, γ, and thermochemical properties; compound **2** ranks second with the highest values in four categories: volume, ΔE, dipole moment, and void volume; and compound **1** ranks third with the highest values in two categories: density and mean polarizability (α_0_). However, in this ranking, in addition to the number of atoms in the compounds, the Z value, which indicates the repeating structure in the unit cell, also plays an important role. Since all three compounds obtained in the present study are transparent, it is recommended that the electrooptical properties of compound **1**, which has the highest chemical reactivity, be investigated by groups working in this field. Comparing the β_0_ values of the obtained compounds with those of urea [CO(NH_2_)_2_] and para-nitroaniline (pNA) compounds, their β_0_ values were found to be 3.34, 3.63, and 52.72 and 79.83, 86.59, and 1258.1 times higher, respectively. This indicates that all three compounds, especially compound **3**, are significant potential candidates for nonlinear optical applications.

## Supplementary materials

Figure S1 An infinite 1D layer in **1**.

Figure S2 The 2D supramolecular network in **1**.

Figure S3 An infinite 1D layer (a) and the 2D supramolecular network (b) in **2**.

Figure S4 The 1D supramolecular network in compound **3**.

Figure S5 Crystal structure of compound **3**, showing the formation of edge-fused $R_{2}^{2}(18)R_{4}^{4}(36)$ rings.

Figure S6 Crystal structure of compound **3**, showing the formation of N–H⋯π interactions.

Figure S7 The charge distribution graphs of the UV transitions with the highest C_i_ coefficients of compounds **1**, **2**, and **3**.

## Figures and Tables

**Table 1:** Elemental analysis results of Werner- and Werner-like-type clathrates.

Compounds; M_r_ (g)	Elemental analysis, (calculated)(%)/found(%)				
	C	H	N	S	Ni
[Ni(NCS)_2_(C_5_H_6_N_2_)_2_(H_2_O)]·(H_2_O); 399.12	(36.11) 35.96	(4.04) 3.95	(21.06) 21.22	(16.07) 15.91	(14.72) 14.83
[Ni(NCS)_2_(C_5_H_6_N_2_)_3_]·(C_2_H_6_); 487.27	(46.83) 46.18	(4.96) 4.76	(23.00) 22.71	(13.16) 12.94	(12.06) 12.12
[Ni(NCS)_2_(C_5_H_6_N_2_)_4_]·2(C_5_H_6_N_2_); 739.54	(51.97) 51.90	(4.91) 4.57	26.52) 26.63	(8.67) 8.43	(7.94) 7.98

**Table 2:** Crystal data and structure refinement parameters of compounds **1**, **2**, and **3**.

Empirical formula	C_12_H_16_N_6_NiO_2_S_2_	C_19_H_24_N_8_NiS_2_	C_32_H_36_N_14_NiS_2_
Formula weight	399.14	487.29	739.58
Crystal system	Monoclinic	Monoclinic	Triclinic
Space group	P2_1_/n	C2/c	P1̄
*a (*Å)	12.0567 (13)	24.915 (4)	10.680 (2)
*b (*Å)	9.8002 (12)	10.9978 (19)	10.8483 (19)
*c (*Å)	14.6438 (18)	17.059 (3)	17.774 (3)
*a* (°)	90.000 (0)	90.000 (0)	101.749 (6)
*b (*º)	94.450 (4)	90.740 (5)	100.958 (5)
g (°)	90.000 (0)	90.000 (0)	105.450 (6)
*V (*Å^3^)	1725.1 (4)	4674.2 (14)	1877.0 (6)
Z	4	8	2
*D*_c_ (g cm^−3^)	1.537	1.385	1.309
θ range (º)	3.0–28.3	2.3–26.9	2.4–21.9
Measured refls.	23287	47332	54290
Independent refls.	3342	4356	6950
*R*_int_	0.055	0.056	0.071
S	1.08	1.03	1.20
R1/wR2	0.072/0.189	0.050/0.159	0.107/0.242
Dr_max_/Dr_min_ (eÅ^−3^)	1.03/−0.69	1.23/–0.70	1.40/−1.17
CCDC	2281577	2337365	2412909

**Table 3:** Selected bond distances and angles for compounds **1**, **2**, and **3** (Å, °).

**Compound 1** Symmetry code: (i) −x+3/2, y+1/2, −z+3/2			
Ni1−N1	2.112 (6)	Ni1−N3	2.133 (5)
Ni1−N5	2.039 (6)	Ni1−N6	2.028 (6)
Ni1−O1	2.123 (6)	Ni1−N4^i^	2.210 (6)
N6−Ni1−N5	175.0 (3)	N6−Ni1−N1	91.2 (2)
N5−Ni1−N1	92.2 (2)	N6−Ni1−O1	89.5 (3)
N5−Ni1−O1	86.7 (2)	N1−Ni1−O1	91.9 (2)
N6−Ni1−N3	91.7 (2)	N5−Ni1−N3	91.9 (2)
N1−Ni1−N3	90.4 (2)	O1−Ni1−N3	177.4 (2)
O1−Ni1−N4^i^	92.5 (2)	N3−Ni1−N4^i^	85.3 (2)
N6−Ni1−N4^i^	86.5 (2)	N5−Ni1−N4^i^	90.4 (2)
**Compound 2** Symmetry code: (i) −x+1/2, y−1/2, −z+1/2			
Ni1−N1	2.188 (3)	Ni1−N3	2.121 (4)
Ni1−N5	2.203 (3)	Ni1−N7	2.051 (4)
Ni1−N8	2.062 (4)	Ni1−N2^i^	2.193 (3)
S1−C16	1.646 (5)	S2−C17	1.634 (4)
N1−C1	1.351 (5)	N1−C5	1.347 (5)
N5−C11	1.345 (5)	N5−C15	1.343 (5)
N3−C6	1.337 (6)	N3−C10	1.333 (6)
N6−C12	1.396 (6)	N2−C4	1.433 (5)
N8−Ni1−N7	178.34 (15)	N5−Ni1–N8	90.90 (13)
N1−Ni1−N5	176.46 (12)	N5−Ni1−N7	90.47 (13)
N1−Ni1−N7	88.30 (13)	N5−Ni1−N3	93.89 (13)
N1−Ni1−N3	89.46 (13)	N5−Ni1−N2^i^	89.69 (12)
S2−C17−N8	178.5 (4)	N7−Ni1−N2^i^	92.42 (13)
S1−C16−N7	178.9 (4)	N1−Ni1−N2^i^	87.05 (12)
N8−Ni1−N2^i^	86.65 (13)	N3−Ni1−N2^i^	174.84 (14)
**Compound 3**			
N1−Ni1	2.154 (7)	N3−Ni1	2.140 (7)
N5−Ni1	2.120 (7)	N7−Ni1	2.159 (7)
N9−Ni1	2.009 (8)	N10−Ni1	2.072 (7)
N9−Ni1−N10	176.1 (3)	N9−Ni1-N5	89.3 (3)
N10−Ni1−N5	88.5 (3)	N9−Ni1-N3	90.6 (3)
N10−Ni1−N3	92.7 (3)	N5−Ni1−N3	90.6 (3)
N9−Ni1−N1	90.7 (3)	N10−Ni1−N1	91.5 (3)
N5−Ni1−N1	179.2 (3)	N3−Ni1−N1	88.6 (3)
N9−Ni1−N7	86.7 (3)	N10−Ni1−N7	90.2 (3)
N5−Ni1−N7	92.4 (3)	N3−Ni1−N7	175.9 (3)

**Table 4:** Hydrogen-bond parameters and N–H⋯π interactions for compounds **1**–**3** (Å, °).

D-H· · ·A	D-H	H⋯A	D⋯A	D-H⋯A
**Compound 1**				
C9—H9⋯S2^ii^	0.93	2.86	3.603 (7)	138
N2—H2B⋯S1^iv^	0.86 (2)	2.87 (8)	3.552 (9)	138
N2—H2A⋯S2^iii^	0.85 (2)	2.80 (2)	3.655 (8)	175
O2—H2C⋯N2^v^	0.83	2.38	3.142 (13)	154
O2—H2D⋯S1^v^	0.83	2.59	3.330 (7)	149
N4—H4A⋯S2^vi^	0.86 (2)	2.77 (3)	3.590 (6)	160
N4—H4B⋯O2^vi^	0.86 (2)	2.11 (2)	2.974 (8)	175
Symmetry codes: (ii) −x+1, −y+1, −z+2; (iii) −x+1/2, y−1/2, −z+3/2; (iv) −x+1, −y, −z+1; (v) −x+1, −y+1, −z+1; (vi) x, y−1, z.				
**Compound 2**				
C1—H1⋯N8	0.93	2.46	3.044 (5)	121
C3—H3⋯S2^ii^	0.93	2.86	3.726 (4)	156
C19—H19C⋯S2	0.96	2.77	3.573 (4)	142
C19—H19B⋯S1^i^	0.96	2.69	3.477 (4)	139
C11—H11⋯N8	0.93	2.49	3.081 (6)	122
C15—H15⋯N7	0.93	2.45	3.054 (6)	122
N2—H2B⋯N6^iii^	0.85	2.55	3.246 (6)	140
N2—H2A⋯S2^iii^	0.86	2.64	3.492 (3)	168
N4—H4B⋯S1^iv^	0.88	2.69	3.569 (6)	177
N6—H6B⋯S1^v^	0.87	2.63	3.490 (4)	168
Symmetry codes: (i) x, 1-y, z-1/2; (ii) −x+1/2, −y+3/2, −z; (iii) x, y+1, z; (iv) −x+1, y, −z+1/2; (v) x, y-1, z.				
**Compound 3**				
N4—H4B⋯S1^i^	0.86	2.79	3.629 (9)	166
N8—H8B⋯S2^ii^	0.86	2.68	3.536 (9)	177
N12—H12B⋯N8	0.86	2.56	3.400 (16)	165
N14—H14A⋯N4^iii^	0.86	2.63	3.44 (2)	155
N2—H2A⋯S1^iv^	0.86	2.76	3.558 (9)	157
N4—H4A⋯Cg1^v^	0.86	2.56	3.382 (3)	161
N8—H8A⋯Cg2^vi^	0.86	2.57	3.413 (2)	166
Symmetry codes: (i) −x+1, −y+1, −z+2; (ii) −x, −y, −z+1; (iii) x, y, z−1; (iv) x, y−1, z; (v) 1-x, -y, 1-z; (vi) 1-x, 1-y, 1-z; Cg1 = N13/C28-C32; Cg2 = N11/C23-C27.				

**Table 5:** The FT-IR absorption wavenumbers of the 3AP ligand molecule in the free state with its compounds and the shift amounts (cm^−1^) due to the formation of compounds in them.

Assignment a	Solid 3AP	1	Δ	2	Δ	3	Δ
ν_as_(N-H)	3373 s	3394 w	21	3432 m	59	3448 w 3420 m	75 47
ν_s_(N-H)	3304 w	3309 vw 3279 w	5 −25	3339 s 3221 m	35 17	3325 s 3209 s	21 −95
ν(C-H)	3066 vw	3145 m	79	3152 m	86	3103 w	37
ν(C-H)	3035 w	3054 w	19	3062 w 3049 w	27 14	3080 w 3067 w 3037 w 3010 w	45 32 2 −25
NH_2_ scissoring	1633 m	1615 s	−18	1630 s	-3	1632 s 1620 s	-1 -13
ν_ring_ + δCH	1584 s	1597 s	13	1595 w 1582 s	11 −2	1598 s 1580 s	24 −4
ν_ring_	1486 s	1489 s	3	1487 s	1	1497 s 1487 s	11 1
δCH + ν_ring_	1433 vs	1450 s	17	1446 s	13	1442 s	9
δCH	1347 w	1341 w	−6	1367 w 1338 m	20 −10	1336 m	−11
ν_C-NH2_ + ν_ring_	1291 s	1302 m	11	1308 m 1289 m	17 −3	1304 s	13
ν_ring_	1260 s	1264 s	4	1262 s	2	1261 s	1
δCH + ν_ring_	1195 w	1235 m	40	1195 s	0	1193 s 1174 m	−2 −21
δCH + ν_ring_+ δCNH	1125 m	1124 w	−1	1140 m	15	1135 m	10
NH_2_ twist + ν_ring_	1091 m	1106 vw	15	1118 s	27	1126 s	35
Ring breath., ν_ring_ + δCNH + δCH	1042 m	1048 s	6	1079 m	37	1084 s 1052 s	42 10
δ_ring_ + ν_ring_	1014 w	1018 s	4	1007 m	−7	1023 s	9
γCH	963 m	950 vw	−13	963 w	0	995 w 977 w	32 14
γCH	908 w	Obs.	–	Obs.	−	954 w 921 w	46 13
γCH	871 m	857 vw	−14	889 m	18	Obs.	−
γCH + γ_ring_ + γ_C-NH2_	797 s	801 s	4	813 m 803 s	16 6	846 w 807 s	49 10
γ_ring_ + γCH	702 s	705 s	3	703 s	1	754 s 702 s	52 0
δ_ring_	623 m	646 m	23	644 s 614 m	19 −11	643 s	20
δ_ring_+NH_2_ wag + γ_C-NH2_	537 w	568 w	21	549 m	12	572 w 547 m	35 10
NH_2_ wag + γ_C-NH2_ + γ_ring_	510 w	532 vw	22	525 w	15	526 w 506 s	16 -4
γ_C-NH2_ +NH_2_ wag + γ_ring_	453 w	Obs.	–	Obs.	−	Obs.	−
∑Δ	−	−	232	-	411	-	566

ν: stretching; δ: out of plane bending; γ: in plane bending; β: out of plane bending; ω: wagging; τ: torsion; ρ: rocking; s: strong; vs: very strong; m: medium; w: weak; vw: very weak; Obs.: Covered by vibration peak of another molecule.

a Taken from [15].

**Table 6:** Fundamental FT-IR vibration wavenumbers (cm^−1^) of solid KNCS and the isothiocyanate group (NCS)^−^ in compounds **1**–**3**.

Assignment a	ν̄^a^	Compounds					
		1	Δ	2	Δ	3	Δ
ν(NC)	2036 s	2108 s	72	2094 s	58	2102 s	66
ν(CS)	740 m	834 m	94	869 s	129	879 m	139
δ(NCS)	484 w 474 s	469 m	-	474 m	-	475 m	-
2δ(NCS)	957 m	909 w	−48	934 w	−23	900 w	−57
∑Δ	-	-	118	-	164	-	148

ν: stretching; δ: out of plane bending; s: strong; m: medium; w: weak;

a Taken from [25].

**Table 7:** Types of Mulliken electric charges of the atoms forming the compounds, in the free state and in the compounds, and their values ​according to the free electron charge (e).

Compound 1								
Atom	ACVFL	ACVC	Atom	ACVFL	ACVC	Atom	ACVFL	ACVC
1 C	0.064406	−0.014254	14 H	0.094471	0.12768	27 S	0.219657	−0.369752
2 H	0.099218	0.129924	15 C	0.00243	−0.094585	28 O	−0.473504	−0.371837
3 C	0.019479	0.128905	16 H	0.104372	0.137543	29 H	0.236752	0.312753
4 C	0.00243	−0.123783	17 C	0.042209	−0.001259	30 H	0.236752	0.320359
5 H	0.093324	0.119068	18 H	0.104372	0.145154	31 N	−0.465967	−0.526075
6 C	−0.181659	−0.100021	19 C	−0.017151	0.110125	32 H	0.209095	0.268372
7 H	0.094471	0.126097	20 C	−0.017151	0.109987	33 H	0.207468	0.276269
8 C	0.042209	−0.013117	21 N	−0.288847	−0.192075	34 N	−0.465967	−0.514328
9 H	0.104372	0.125019	22 N	−0.288847	−0.158376	35 H	0.209095	0.274506
10 C	0.064406	−0.011632	23 N	−0.202506	−0.221834	36 H	0.207468	0.272656
11 H	0.099218	0.146507	24 N	−0.202506	−0.229117	37 O	−0.473504	−0.507551
12 C	0.019479	0.122385	25 Ni	2.000000	0.146843	38 H	0.236752	0.274986
13 C	0.019479	−0.106956	26 S	0.219657	−0.385557	39 H	0.236752	0.266971
**Compound 2**								
**Atom**	**ACVFL**	**ACVC**	**Atom**	**ACVFL**	**ACVC**	**Atom**	**ACVFL**	**ACVC**
1 C	0.04221	0.012241	19 C	0.06441	−0.00962	37 S	0.21966	−0.18912
2 H	0.10437	0.122997	20 H	0.09922	0.118297	38 N	−0.466	−0.534
3 C	−0.1817	−0.07918	21 C	0.01948	0.175615	39 H	0.2091	0.256878
4 H	0.09447	0.107943	22 C	0.00243	−0.10167	40 H	0.20747	0.244907
5 C	0.00243	−0.09501	23 H	0.09332	0.097489	41 N	−0.466	−0.52096
6 H	0.09332	0.10313	24 C	−0.1817	−0.0789	42 H	0.2091	0.249097
7 C	0.01948	0.143428	25 H	0.09447	0.108406	43 H	0.20747	0.23855
8 C	0.06441	0.004087	26 C	0.04221	0.013132	44 N	−0.466	−0.58501
9 H	0.09922	0.128954	27 H	0.10437	0.124947	45 H	0.2091	0.29681
10 C	0.06441	0.022487	28 C	−0.0172	−0.00226	46 H	0.20747	0.246876
11 H	0.09922	0.126691	29 C	−0.0172	−0.00446	47 C	−0.2851	−0.29869
12 C	0.01948	0.14662	30 N	−0.2888	−0.22969	48 H	0.09502	0.08864
13 C	0.00243	−0.08233	31 N	−0.2888	−0.25745	49 H	0.09502	0.101798
14 H	0.09332	0.110761	32 N	−0.2888	−0.23012	50 H	0.09502	0.111841
15 C	−0.1817	−0.0763	33 N	−0.2025	−0.42205	51 C	−0.2851	−0.29481
16 H	0.09447	0.119212	34 N	−0.2025	−0.42097	52 H	0.09502	0.098836
17 C	0.04221	0.041683	35 Ni	2.00000	0.633964	53 H	0.09502	0.087967
18 H	0.10437	0.12824	36 S	0.21966	−0.19852	54 H	0.09502	0.098586
**Compound 3**								
**Atom**	**ACVFL**	**ACVC**	**Atom**	**ACVFL**	**ACVC**	**Atom**	**ACVFL**	**ACVC**
1 C	0.042209	−0.001300	30 C	−0.181659	−0.104555	59 H	0.207468	0.305486
2 H	0.104372	0.142593	31 H	0.094471	0.132542	60 C	0.042209	−0.055733
3 C	−0.181659	−0.097383	32 C	0.002430	−0.114575	61 H	0.104372	0.111627
4 H	0.094471	0.142419	33 H	0.093324	0.127858	62 C	−0.181659	−0.110200
5 C	0.002430	−0.109711	34 C	0.019479	0.129800	63 H	0.094471	0.116177
6 H	0.093324	0.136779	35 C	0.064406	−0.033400	64 C	0.002430	−0.140882
7 C	0.019479	0.126675	36 H	0.099218	0.126766	65 H	0.093324	0.115104
8 C	0.064406	−0.010745	37 C	−0.017151	0.056099	66 C	0.019479	0.125106
9 H	0.099218	0.143509	38 C	−0.017151	0.052263	67 C	0.064406	−0.054181
10 C	0.042209	−0.022169	39 N	−0.288847	−0.165016	68 H	0.099218	0.111100
11 H	0.104372	0.130138	40 N	−0.288847	−0.174563	69 N	−0.288847	−0.251324
12 C	−0.181659	−0.107261	41 N	−0.465967	−0.516785	70 N	−0.465967	−0.517937
13 H	0.094471	0.131961	42 H	0.209095	0.291634	71 H	0.209095	0.273789
14 C	0.002430	−0.117055	43 H	0.207468	0.293244	72 H	0.207468	0.268480
15 H	0.093324	0.128202	44 N	−0.288847	−0.156784	73 C	0.042209	−0.065486
6 C	0.019479	0.135589	45 N	−0.288847	−0.176925	74 H	0.104372	0.102160
17 C	0.064406	−0.028588	46 N	−0.465967	−0.521833	75 C	−0.181659	−0.119027
18 H	0.099218	0.128367	47 H	0.209095	0.292807	76 H	0.094471	0.114028
19 C	0.042209	−0.008448	48 H	0.207468	0.296012	77 C	0.002430	−0.133321
20 H	0.104372	0.141329	49 N	−0.202506	−0.221729	78 H	0.093324	0.110410
21 C	−0.181659	−0.107078	50 N	−0.202506	−0.216163	79 C	0.019479	0.143050
22 H	0.094471	0.142065	51 Ni	2.000000	0.231412	80 C	0.064406	−0.084771
23 C	0.002430	−0.111970	52 S	0.219657	−0.571396	81 H	0.099218	0.110582
24 H	0.093324	0.136975	53 S	0.219657	−0.569323	82 N	−0.288847	−0.247504
25 C	0.019479	0.135994	54 N	−0.465967	−0.529785	83 N	−0.465967	−0.521767
26 C	0.064406	−0.015245	55 H	0.209095	0.285172	84 H	0.209095	0.295587
27 H	0.099218	0.130560	56 H	0.207468	0.288356	85 H	0.207468	0.296059
28 C	0.042209	−0.020540	57 N	−0.465967	−0.529407			
29 H	0.104372	0.128967	58 H	0.209095	0.297034			

ACVFL: Atomic charge value in free ligand, ACVC: Atomic charge value in the compound.

**Table 8:** The HOMO, LUMO and chemical efficiency values in (eV) units of compounds.

Chemical efficiency values	1	2	3
E_HOMO_ (-I)	−4.615	−1.913	−3.834
E_LUMO_ (-A)	−3.298	4.082	−2.125
ΔE = E_LUMO_ - E_HOMO_	1.317	5.995	1.709
χ	3.957	−1.084	2.980
μ	−3.957	1.084	−2.980
η	0.659	2.997	0.854
S (eV)^−1^	0.759	0.166	0.585
ω	11.885	0.1961	5.195

**Table 9:** Values of some NLO properties of compounds in esu units.

Parameters	Compounds		
	1	2	3
μ (D)	7.422645	10.161011	8.216719
Δα	7.422645×10^−23^	10.161011×10^−23^	8.216719×10^−23^
α_0_	−2.198653×10^−23^	−2.741972×10^−23^	−4.665998×10^−23^
β_0_	1.237254×10^−30^	1.342080×10^−30^	1.950060×10^−29^
γ	−2.065008×10^−36^	−3.041814×10^−36^	−1.533659×10^−35^

**Table 10:** Some thermochemical data of the compounds.

Thermochemical properties	Components	1	2	3
E (kcal/mol)	Electronic	0	0	0
	Translational	0.889	0.889	0.889
	Rotational	0.899	0.889	0.889
	Vibrational	164.274	300.309	467.221
	Total	166.062	302.086	468.998
Heat capacity at constant volume C_V_ (cal/mol-Kelvin)	Electronic	0	0	0
	Translational	2.981	2.981	2.981
	Rotational	2.981	2.981	2.981
	Vibrational	41.028	67.826	112.225
	Total	46.99	73.787	118.187
Entropy S (cal/mol-Kelvi)	Electronic	0	0	0
	Translational	43.594	44.431	45.677
	Rotational	35.464	36.864	40.766
	Vibrational	42.28	71.793	136.614
	Total	122.715	153.089	223.057
Zero-point vibrational energy E_v0_	(Joules/mol)	659795.6	1210291	1876392
	(kcal/mol)	157.695	289.267	448.468
Rotational constants (GHz)	A	0.25135	0.15337	0.06545
	B	0.17143	0.09332	0.01978
	C	0.11078	0.08149	0.01775

**Table 11:** List of some properties of the five theoretical excitation states that make the greatest contribution to the UV–Vis excitation states of the compounds.

Compounds	Excitation states	Excitation energy (eV)	Wavelength (nm)	Oscillation strengths (f)	Assignments From → to	Coefficients (C_i_)	Contribution (%)
**1**	12	26.340	470.70	0.0005	80 → 89	0.65063	0.8466387
	13	27.809	445.85	0.0013	79 → 89	0.69260	0.9593895
	15	31.745	390.56	0.0006	78 → 89	0.60611	0.7347386
	18	42.876	289.17	0.0007	88 → 90	0.50073	0.5014610
	20	46.301	267.78	0.0093	86 → 90	0.57091	0.6518764

**2**	12	28.122	440.88	0.0029	104 → 113	0.64632	0.8354590
	13	31.321	395.84	0.0001	102 → 113	0.57653	0.6647736
	18	42.914	288.91	0.0063	96 → 113	0.64784	0.8393933
	19	44.673	277.54	0.0254	95 → 113	0.62808	0.7889689
	20	45.493	272.54	0.0053	112 → 114	0.65180	0.8496864

**3**	11	20.472	605.64	0.0022	169 → 179	0.67574	0.9132490
	12	20.710	598.66	0.0021	168 → 179	0.64446	0.8306573
	15	23.939	517.92	0.0072	167 → 179	0.57523	0.6617791
	17	34.900	355.26	0.0006	178 → 180	0.67110	0.9007504
	18	35.306	351.17	0.0002	178 → 181	0.67130	0.9012873

**Table 12:** The NMR theoretical isotropic and anisotropic chemical and magnetic shielding values calculated for compounds **1**–**3** in water solvent at room temperature.

Atoms	Elements	Degeneracy	Chemical and magnetic shielding values (ppm)		
			Isotropic		Anisotropic
			Average	Shielding value corrected to reference matter	
**Compound 1**					
25	Ni	1	−14021.469	−	10120.7885
11;7	H	2	27.1485	4.7336	6.8417;6.6369
29	H	1	38.403	−6.5209	30.9475
27	S	1	50.397	−	40.185
26	S	1	51.599	−	40.336
21	N	1	96.24	−	389.7807
12	C	1	112.728	69.7376	134.7547
4	C	1	139.662	42.8036	143.1016
22	N	1	146.894	−	272.9847
31	N	1	287.232	−	43.0804
37	O	1	441.543	−	43.3475
28	O	1	532.089	−	312.9287
**Compound 2**					
35	Ni	1	−14495.858	−	9248.1576
18	H	1	26.088	5.7941	8.5601
16;6	H	2	27.4675	4.4146	9.3493;6.1915
53;52;48;49	H	4	36.742	−4.8599	12.3877;16.1262;14.5584;14.8755
37	S	1	50.838	−	40.7149
36	S	1	51.47	−	40.7981
31	N	1	96.316	162.084	380.8157
7	C	1	109.495	72.9706	136.1166
30	N	1	135.285	123.115	284.8512
51	C	1	242.953	−60.4874	17.5757
34	N	1	267.704	−9.304	111.3195
41	N	1	287.137	−28.737	37.6645
**Compound 3**					
51	Ni	1	−13530.475	−	15588.989
82	N	1	−18.378	240.022	574.7029
11	H	1	26.702	5.1801	8.6854
36;18	H	2	27.4015	4.4806	8.4052;9.2331
42;59;58	H	3	34.346	−2.4639	18.8402;17.9093;17.2330
53	S	1	60.712	−	28.1172
45	N	1	108.427	149.973	359.977
23	C	1	139.289	43.1766	142.9755
44	N	1	139.399	119.001	300.7229
21	C	1	139.58	42.8856	152.1215
39	N	1	141.11	117.29	308.6448
64	C	1	144.041	38.4246	135.0149
70;83	N	2	283.676	−25.276	66.1357;44.8569

**Table 13:** Percentage contributions of various intermolecular contacts of the compounds.

Intermolecular contacts	Compounds			Intermolecular contacts	Compounds		
	1	2	3		1	2	3
C⋯C	1.7	4.3	2.3	H⋯O/O⋯H	2.5	-	-
C⋯H/H⋯C	24.8	18.0	22.9	H⋯S/S⋯H	28.5	22.2	12.9
C⋯N/N⋯C	1.8	1.7	1.6	N⋯N	0.3	0.4	0.0
C⋯O/O⋯C	1.2	-	-	N⋯Ni/Ni⋯N	1.6	1.5	0.0
C⋯S/S⋯C	2.9	1.7	0.0	N⋯O/O⋯N	0.4	-	-
H⋯H	27.2	39.6	46.8	N⋯S/S⋯N	0.1	0.2	0.4
H⋯N/N⋯H	7.3	9.2	12.2	S⋯S	0.1	0.7	0.9
H⋯Ni/Ni⋯H	0.5	0.5	0.0				

**Table 14:** General characteristics of the voids in the unit cells of the compounds.

Compounds	Volume (Å^3^)	RVVTV (%)	Void area (Å^2^)	Globularity	Asphericity
**1**	180.71	10.48	582.44	0.265	0.079
**2**	637.38	13.64	1633.69	0.219	0.180
**3**	301.70	16.07	842.07	0.258	0.159

RVVTV (%). Ratio of void volume to total volume (%).
