# Supplementary figures and images for: Synthesis of Werner- and Werner-like-type clathrates, their characterization, and theoretical calculation of their properties
Source: Turk J Chem. 2026 Mar 25;50(2):146–72. doi: 10.55730/1300-0527.3787 (PMC13189376; doi:10.55730/1300-0527.3787)

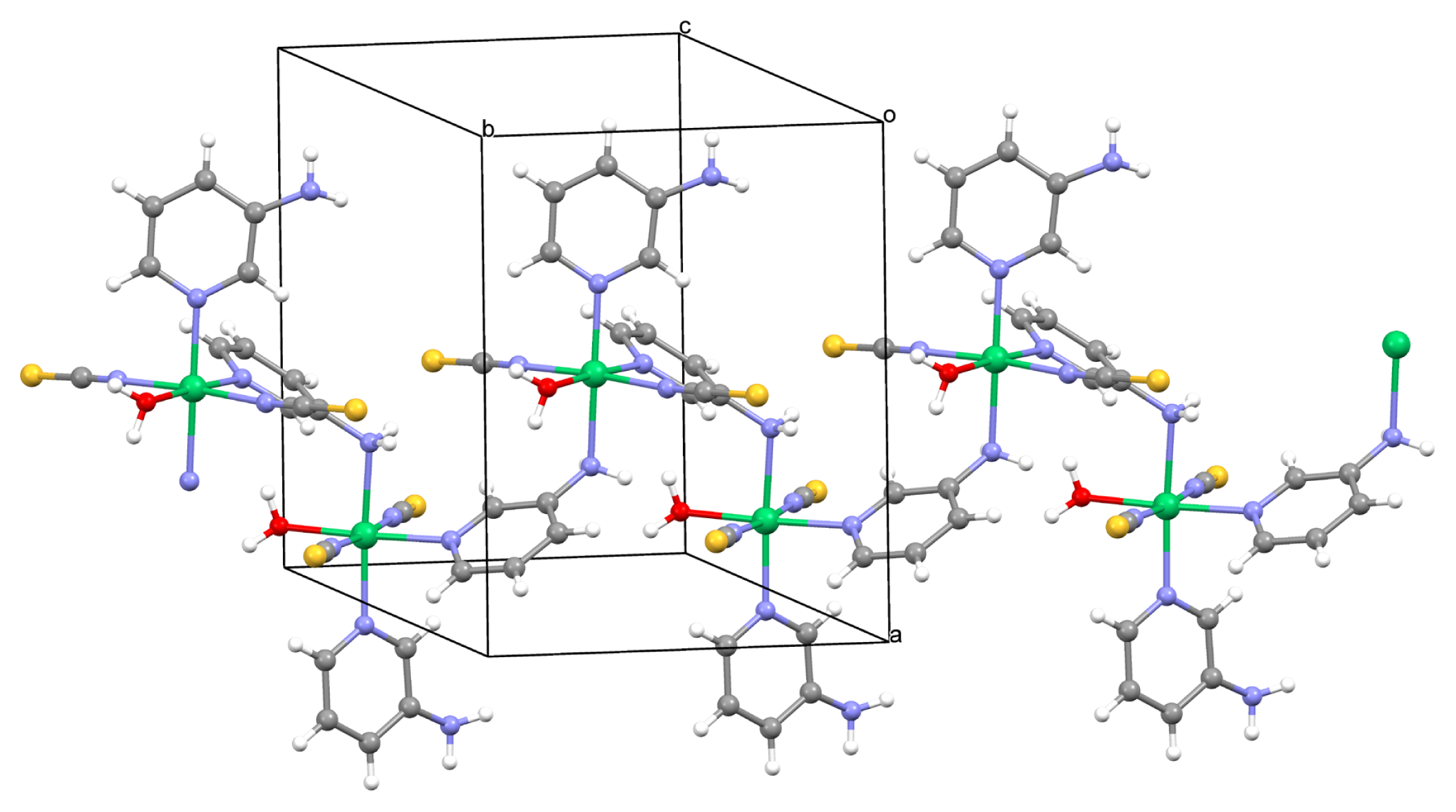

Supplement: Figure S1 — An infinite 1D layer in 1. [file tjc-50-02-146s1.tif]

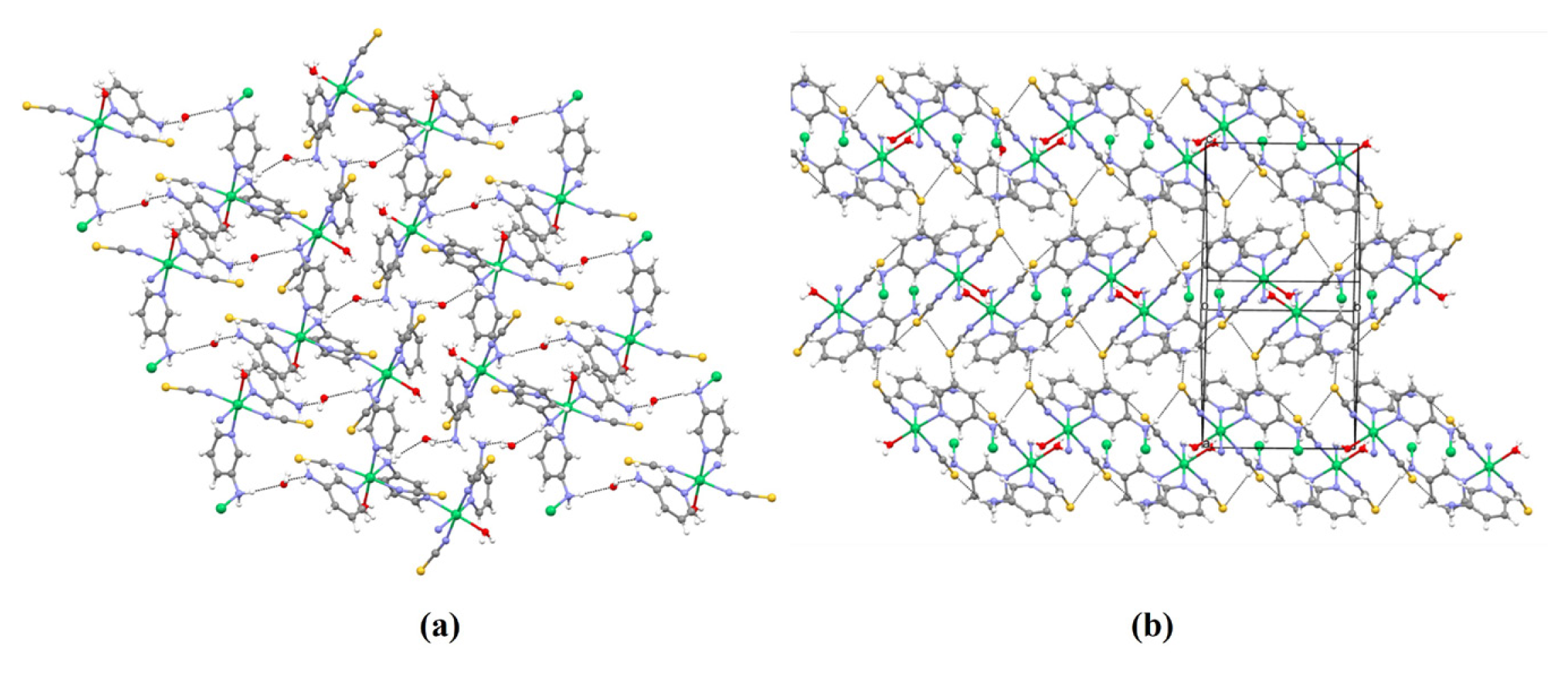

Supplement: Figure S2 — The 2D supramolecular network in 1. [file tjc-50-02-146s2.tif]

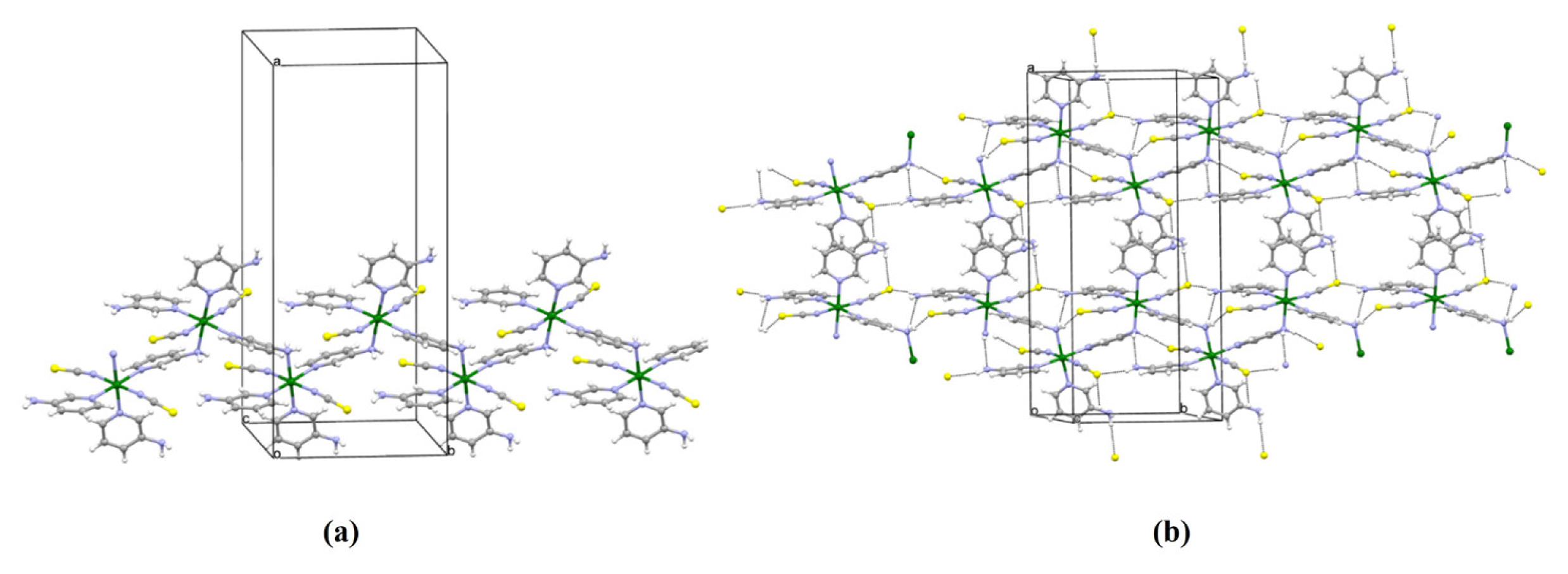

Supplement: Figure S3 — An infinite 1D layer (a) and the 2D supramolecular network (b) in 2. [file tjc-50-02-146s3.tif]

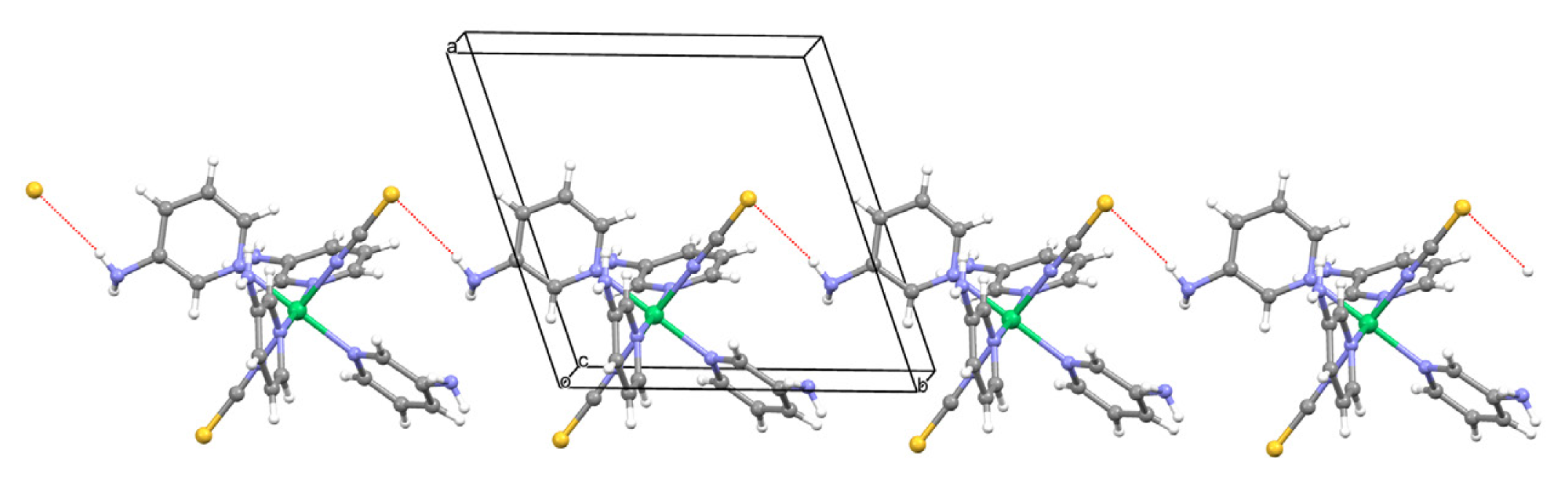

Supplement: Figure S4 — The 1D supramolecular network in compound 3. [file tjc-50-02-146s4.tif]

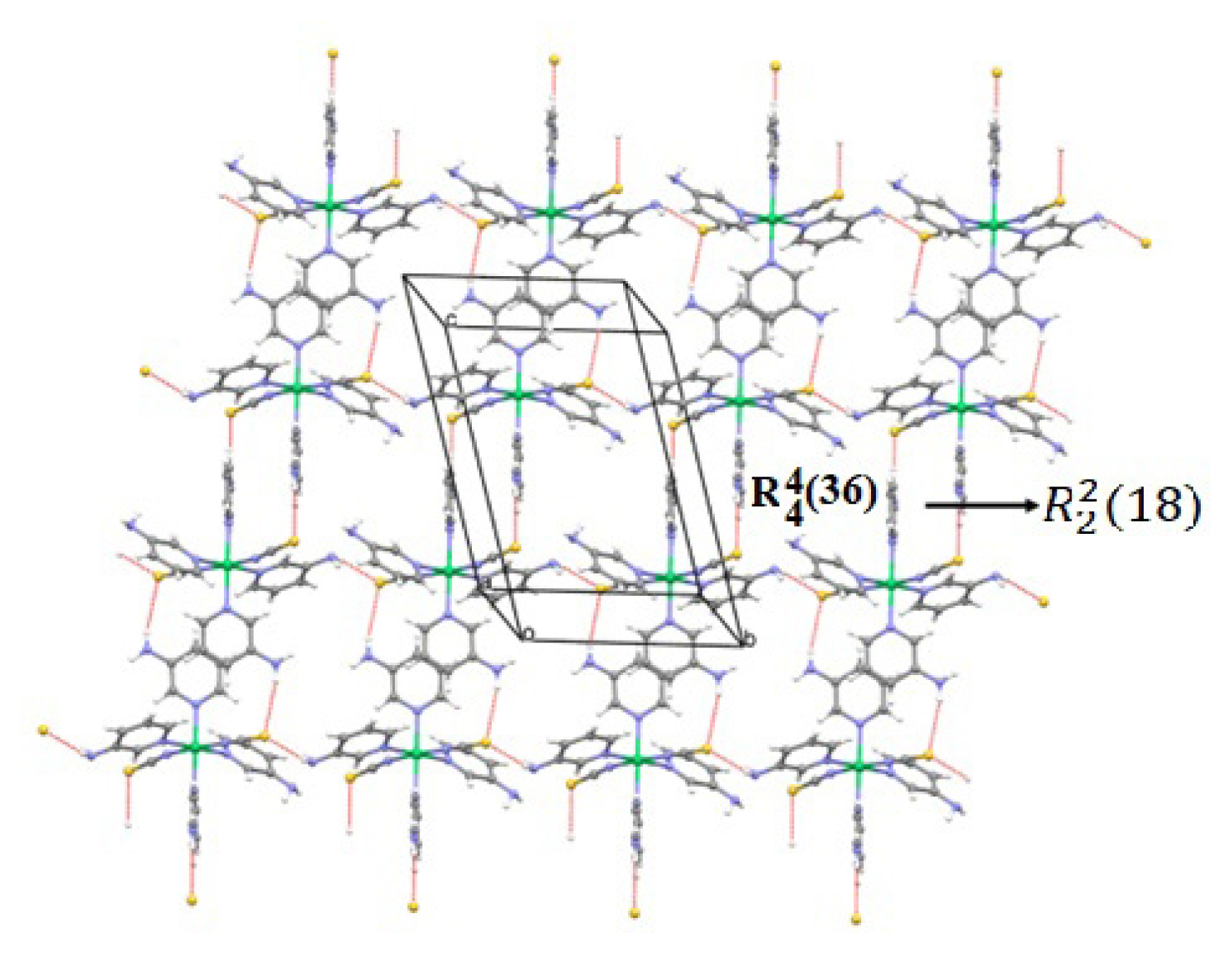

Supplement: Figure S5 — Crystal structure of compound 3, showing the formation of edge-fused R22(18)R44(36) rings. [file tjc-50-02-146s5.tif]

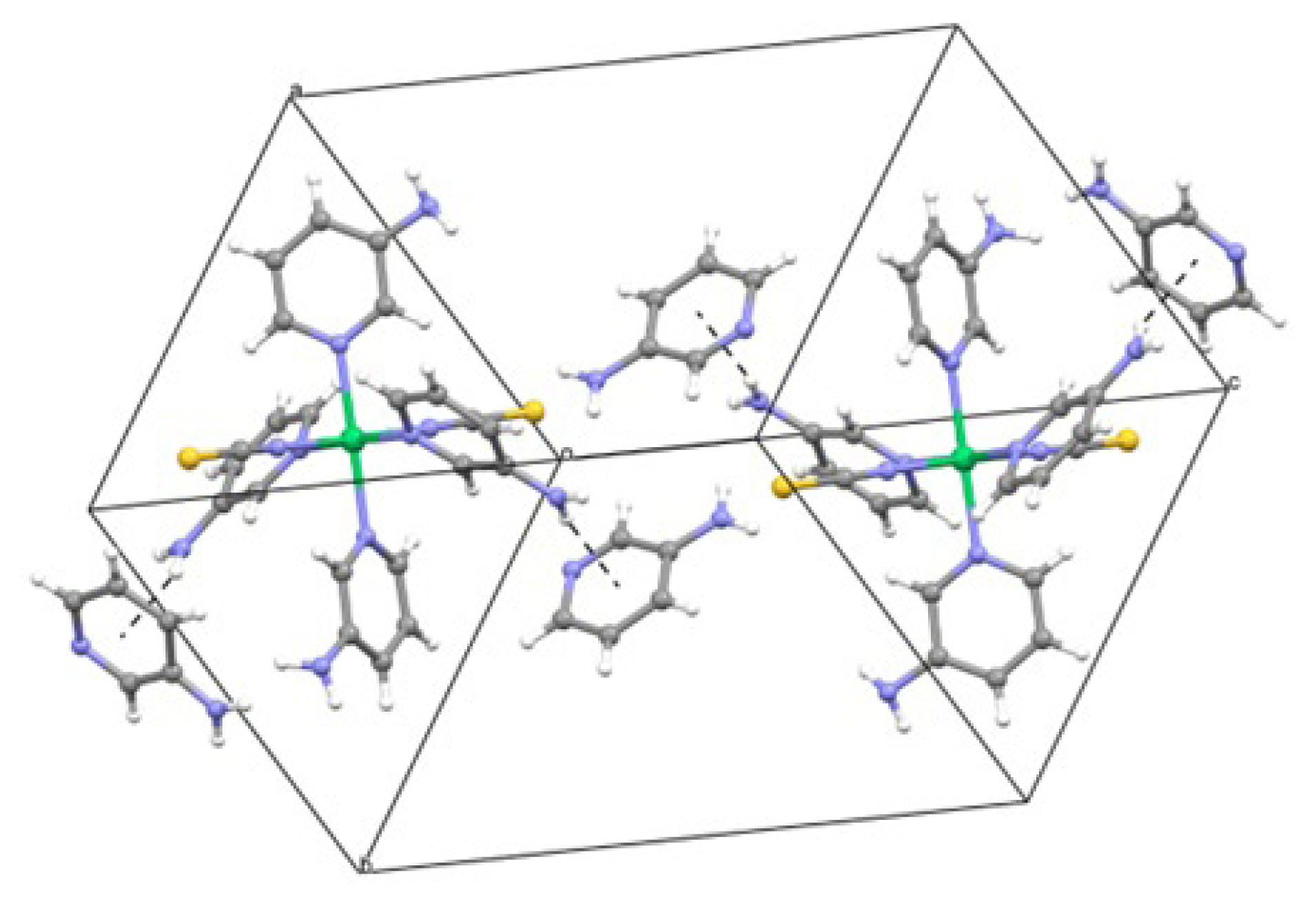

Supplement: Figure S6 — Crystal structure of compound 3, showing the formation of N–H⋯π interactions. [file tjc-50-02-146s6.tif]

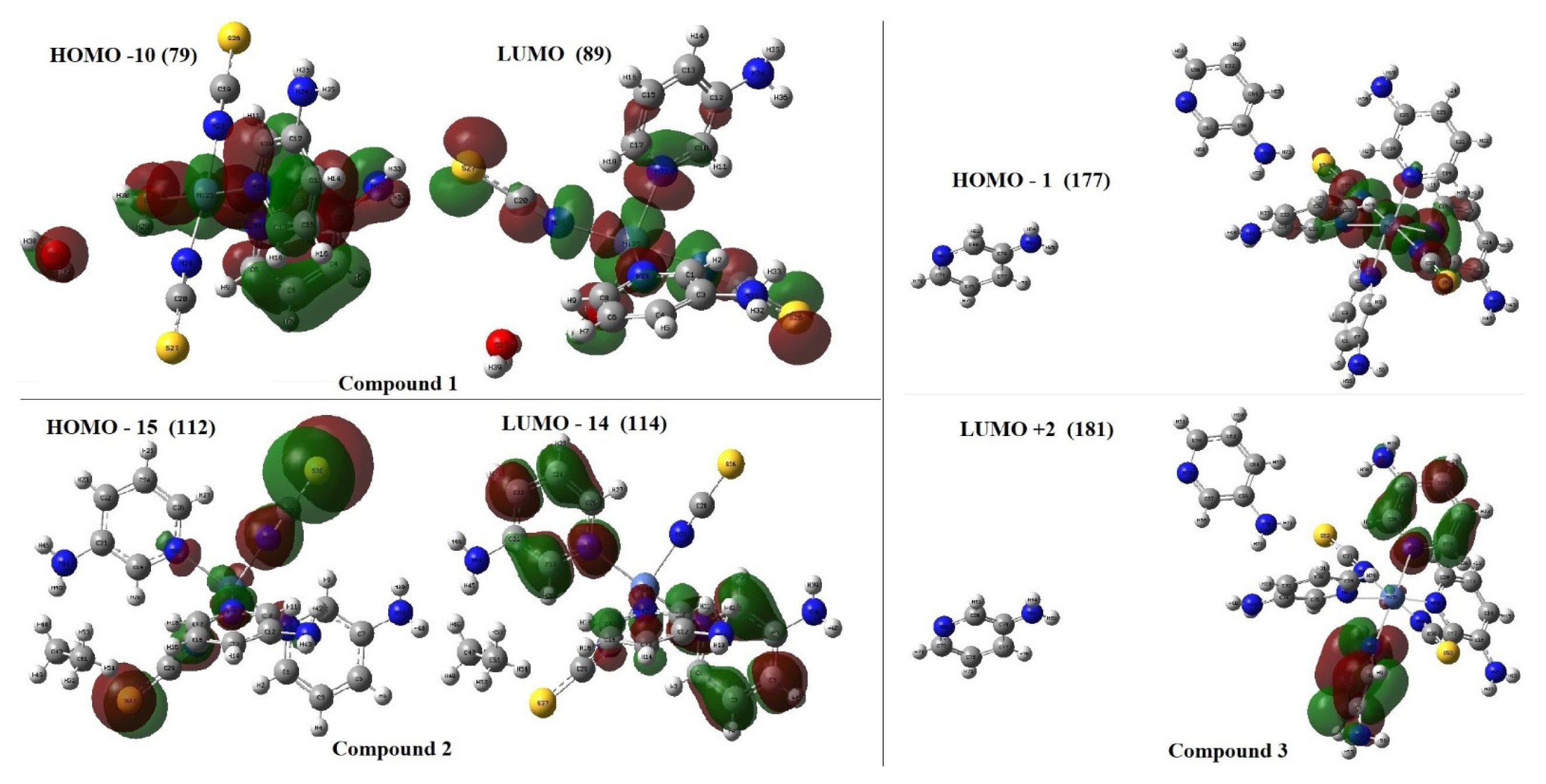

Supplement: Figure S7 — The charge distribution graphs of the UV transitions with the highest Ci coefficients of compounds 1, 2, and 3. [file tjc-50-02-146s7.tif]
